# Transcriptome and excretory–secretory proteome of infective-stage larvae of the nematode *Gnathostoma spinigerum* reveal potential immunodiagnostic targets for development

**DOI:** 10.1051/parasite/2019033

**Published:** 2019-06-05

**Authors:** Supaporn Nuamtanong, Onrapak Reamtong, Orawan Phuphisut, Palang Chotsiri, Preeyarat Malaithong, Paron Dekumyoy, Poom Adisakwattana

**Affiliations:** 1 Department of Helminthology, Faculty of Tropical Medicine, Mahidol University Bangkok 10400 Thailand; 2 Department of Molecular Tropical Medicine and Genetics, Faculty of Tropical Medicine, Mahidol University Bangkok 10400 Thailand; 3 Mahidol-Oxford Tropical Medicine Research Unit, Mahidol University Bangkok 10400 Thailand

**Keywords:** *Gnathostoma spinigerum*, Advanced third stage larva, Transcriptomics, Excretory–secretory proteins, Proteomics, Serpin, Immunodiagnosis

## Abstract

*Background*: *Gnathostoma spinigerum* is a harmful parasitic nematode that causes severe morbidity and mortality in humans and animals. Effective drugs and vaccines and reliable diagnostic methods are needed to prevent and control the associated diseases; however, the lack of genome, transcriptome, and proteome databases remains a major limitation. In this study, transcriptomic and secretomic analyses of advanced third-stage larvae of *G. spinigerum* (aL3Gs) were performed using next-generation sequencing, bioinformatics, and proteomics. *Results*: An analysis that incorporated transcriptome and bioinformatics data to predict excretory–secretory proteins (ESPs) classified 171 and 292 proteins into classical and non-classical secretory groups, respectively. Proteins with proteolytic (metalloprotease), cell signaling regulatory (i.e., kinases and phosphatase), and metabolic regulatory function (i.e., glucose and lipid metabolism) were significantly upregulated in the transcriptome and secretome. A two-dimensional (2D) immunomic analysis of aL3Gs-ESPs with *G. spinigerum*-infected human sera and related helminthiases suggested that the serine protease inhibitor (serpin) was a promising antigenic target for the further development of gnathostomiasis immunodiagnostic methods. *Conclusions*: The transcriptome and excretory–secretory proteome of aL3Gs can facilitate an understanding of the basic molecular biology of the parasite and identifying multiple associated factors, possibly promoting the discovery of novel drugs and vaccines. The 2D-immunomic analysis identified serpin, a protein secreted from aL3Gs, as an interesting candidate for immunodiagnosis that warrants immediate evaluation and validation.

## Abstract (fr)

*Contexte* : *Gnathostoma spinigerum* est un nématode parasite nuisible qui provoque une morbidité et une mortalité graves chez les humains et les animaux. Des médicaments et des vaccins efficaces et des méthodes de diagnostic fiables sont nécessaires pour prévenir et contrôler les maladies associées. Cependant, l’absence de bases de données sur le génome, le transcriptome et le protéome reste une limitation majeure. Dans cette étude, des analyses transcriptomiques et sécrétomiques de larves avancées au troisième stade de *G. spinigerum* (aL3G) ont été effectuées par des méthodes de nouvelle génération de séquençage, bioinformatique et protéomique. *Résultats* : Une analyse incorporant des données de transcriptome et de bioinformatique pour prédire les protéines excréto-sécrétoires (ESP) a classé respectivement 171 et 292 protéines en groupes de sécrétions classiques et non classiques. Les protéines protéolytiques (métalloprotéases), régulatrices de la signalisation cellulaire (kinases et phosphatases) et régulatrices métaboliques (métabolisme du glucose et des lipides) étaient régulées à la hausse dans le transcriptome et le sécrétome. Une analyse immunomique bidimensionnelle (2D) des aL3Gs-ESP avec des sérums humains infectés par *G. spinigerum* et des helminthiases apparentées a suggéré que l’inhibiteur de la sérine protéase (serpine) était une cible antigénique prometteuse pour le développement ultérieur de méthodes immunodiagnostiques de la gnathostomose. *Conclusions* : Le transcriptome et le protéome excréto-sécrétoire des aL3G peuvent faciliter la compréhension de la biologie moléculaire de base du parasite et l’identification de multiples facteurs associés, favorisant éventuellement la découverte de nouveaux médicaments et vaccins. L’analyse 2D-immunomique a identifié la serpine, une protéine sécrétée par les aL3G, comme un candidat intéressant pour l’immunodiagnostic, qui mérite une évaluation et une validation immédiates.

## Introduction

Gnathostomiasis is an important foodborne parasitic zoonosis caused by nematodes of the genus *Gnathostoma* Owen, 1836. Until now, 23 species have been described in this genus that distribute in various regions of the world [10, 53]. Of these, there are six species including *G. spinigerum*, *G. binucleatum*, *G. doloresi*, *G. hispidum*, *G. malaysiae*, and *G. nipponicum* that have been reported to infect humans [10]. Outbreaks of human gnathostomiasis have frequently occurred in Asia and Central America [1, 17], and sporadic cases reported in several countries have been associated with travelers who returned home after visiting endemic areas [31, 43]. In Southeast Asia, *G. spinigerum* is considered a major gnathostomiasis-causing species in humans. *G. spinigerum* is classified in Phylum Nematoda, Order Rhabditida, Family Gnathostomatidae, and Genus *Gnathostoma.* Humans are infected by consumption of raw or improperly cooked fishes, amphibians, and eels that harbor infective (advanced third-stage) larvae. Among these, swamp eels (*Monopterus albus*) are a common source of human gnathostomiasis [16].

Clinical manifestations observed during *G. spinigerum* infection include intermittent subcutaneous or cutaneous migratory swelling with peripheral eosinophilia. The migration of parasites to visceral organs, such as the brain, spinal cord, and eyes, can potentially cause severe diseases [7, 15, 65]. Clinical signs and symptoms, with a history of ingesting raw or improperly cooked secondary intermediate or paratenic hosts, have been used to guide the diagnosis of gnathostomiasis. Alternatively, immunodiagnostic methods have been developed and used to support the diagnosis of this disease. Currently, immunoblotting to detect the 24-kDa crude worm antigen (CWA) expressed by advanced third-stage larvae of *G. spinigerum* (aL3Gs) is a reliable immunodiagnostic technique that provides high sensitivity and specificity rates (80%–90%) [28, 35]. However, the identification of additional diagnostic candidates may help to increase the reliability and effectiveness of immunodiagnosis.

Parasitic helminths release excretory–secretory products (ESPs) that are indispensable to nutrient uptake, tissue penetration, immune invasion, host–parasite interactions, and other processes in all life stages [13, 57]. Moreover, ESPs have been used as diagnostic tools as they are released into the blood circulation and can induce antibody production in the infected host. Accordingly, ESPs could be used to develop immunoassays to detect circulating antigens or antibodies [3, 57]. In the context of a *G. spinigerum* infection, the identification of a specific IgE against ESPs from aL3Gs could improve the power of diagnostic methods and yield improved sensitivity and specificity relative to CWA [54]. In a mouse model, an early infection could be identified by using a two-site enzyme-linked immunosorbent assay to detect circulating aL3G antigens [40]. However, information regarding ESPs of aL3Gs and the identification of novel candidates for immunodiagnostic methods remain limited and require further analysis.

Therefore, this study aimed to use an integrative approach to identify a novel target derived from ESPs of aL3Gs. A global cDNA-transcribed library of aL3Gs was constructed using next-generation sequencing (NGS), and potential ESPs were predicted through a bioinformatics analysis. A two-dimensional (2D) gel electrophoresis (2DE) with an incorporated immunomic assay was performed. Subsequently, the peptide masses were evaluated using mass spectrometry, and the in-house cDNA-transcribed library was used to search for a specific protein. In the future, the study findings may have a remarkable impact on the development of reliable immunodiagnostics, as well as novel therapies against gnathostomiasis.

## Materials and methods

### Parasite

The aL3Gs were obtained from the livers of naturally infected eels using an acid-pepsin digestion technique [44]. In summary, the eel livers were chopped, digested in 1% acid-pepsin at 37 °C for 2 h in a water bath with frequent stirring, and washed several times with tap water via a simple sedimentation technique. The worms were isolated and identified using a dissecting microscope and washed several times with normal saline solution (0.85% NaCl), followed by distilled water. Approximately 20 worms were pooled into a microfuge tube and stored at −80 °C until further use.

### Transcriptome analysis using the Illumina HiSeq platform

Total RNA was extracted from aL3Gs using TRIZOL^TM^ reagent (Invitrogen, Carlsbad, CA, USA) according to the manufacturer’s instructions and quantified and qualified using an Agilent 2100 Bioanalyzer (Agilent Technologies, Palo Alto, CA, USA), NanoDrop spectrophotometer (Thermo Fisher Scientific, Inc., Wilmington, DE, USA) Supplementary Fig. S1), and 1% agarose gels. One microgram of total RNA with a RIN value >7 was used to prepare the library as follows [55]. The library was constructed using the NEBNext^®^ UltraTM RNA Library Prep Kit for Illumina^®^ (New England Biolabs [NEB], Ipswich, MA, USA) according to the manufacturer’s protocol. Poly(A) mRNA isolation was performed using the NEBNext Poly(A) mRNA Magnetic Isolation Module (NEB). mRNA fragmentation and priming were performed using the NEBNext First Strand Synthesis Reaction Buffer and NEBNext Random Primers. First-strand cDNA was then synthesized from mRNA using ProtoScript II Reverse Transcriptase, and second-strand cDNA was synthesized using Second Strand Synthesis Enzyme Mix. Double-stranded cDNA was purified using AxyPrep Mag PCR Clean-up (Axygen Biosciences, Union City, CA, USA) and treated with End Prep Enzyme Mix to repair both ends and add a dA-tail in a single reaction, followed by T-A ligation to add adaptors to both ends. The adaptor-ligated DNA fragments were subjected to size selection using AxyPrep Mag PCR Clean-up (Axygen), which recovered fragments measuring approximately 360 bp. Each sample was then amplified by PCR for 11 cycles using P5 and P7 primers; both primers carry sequences that can anneal to the flow cell to enable bridge PCR, while the P7 primer carries a six-base index that enables multiplexing. The PCR products were cleaned using AxyPrep Mag PCR Clean-up (Axygen), validated using an Agilent 2100 Bioanalyzer (Agilent Technologies, Palo Alto, CA, USA), and quantified using a Qubit 2.0 Fluorometer (Invitrogen, Carlsbad, CA, USA). Next, libraries with different indices were multiplexed and loaded on an Illumina HiSeq instrument, according to the manufacturer’s instructions (Illumina, San Diego, CA, USA). Sequencing was performed using a 2 × 150-bp paired-end (PE) configuration; here, image analysis and base calling were conducted on a HiSeq instrument equipped with HiSeq Control Software (HCS) + RTA 2.7 (Illumina).

Sequencing quality was processed according to the following steps: first, the read quality was assessed using FastQC v0.11.5 software. Nucleotide read quality estimates were produced using the PHRED scale. Second, sequencing adapters were trimmed from the raw reads with inexact matches using Trimmomatic v0.32 software; at the Q20 quality level, a maximum of two mismatches were allowed. Third, the first three nucleotides were trimmed from each read. Fourth, low-quality bases less than Q30 from each end were further trimmed using the running average algorithm. Finally, the resulting sequences with lengths of at least 30 bp were selected for the analysis. The quality of the reads was assessed with FastQC v0.11.5 software.

### Bioinformatics of the aL3Gs transcriptome

High-quality sequences (Q30 or higher) were selected for the assembly. The Bridger program, *de novo* transcriptome assembler, was used to produce contig sequences without reference genome [11]. Subsequently, the assembled contigs were arranged in groups that each contained several tentative transcript variants originating from the same locus.

To annotate the assembled transcripts, two complementary strategies were applied. First, the BLASTX algorithm (BLAST v.2.2.31) [9] was used to align the assembled transcript sequences directly against all Metazoa protein sequences in the UniProt database. Second, each transcript was translated into all possible open reading frames (ORF) using FragGeneScan-plus [32], and each ORF was then aligned against the Conserved Domain Database (CDD) using RPS-BLAST (Reverse Position-Specific BLAST; https://www.ncbi.nlm.nih.gov/Structure/cdd/wrpsb.cgi) [41]. If the transcripts were not functionally annotated sufficiently well in this step, the best UniProt alignment was selected as representative and was complemented with the best CDD annotation according to the blast Bit score and the *E*-value. A threshold of *E*-value < 10^−5^ was applied in functional prediction analysis. Additionally, the transcripts were categorized by homology with conserved domains and with protein families via annotation with the gene ontology (GO) database. The aL3Gs transcripts were then assigned into three GO categories: molecular function, cellular component, and biological process [2].

### *In silico* analysis of Excretory–Secretory Proteins

Potential ESPs were predicted from the aL3Gs transcriptome using methods published by Garg and Ranganathan [22, 23] and Pan *et al.* [46], with some modification. In summary, the classifications of all possible proteins (*N* = 35,850) annotated using the CDD database as classical and non-classical secreted proteins were predicted using SignalP 4.1 [5] and SecretomeP 2.0 [4], respectively. TargetP 1.1 was used to predict mitochondrial target signals [21]; THHMM 2.0 was used to predict transmembrane helices [20]; we then used the results of TargetP 1.1 and THHMM 2.0 to exclude mitochondrial and transmembrane proteins from the potential ESPs, respectively. The ESP prediction pipeline is summarized in Supplementary Figure S2. Finally, both potential classical and non-classical ESPs were annotated against the GO database.

### Excretory–Secretory Proteome

ESPs of aL3Gs were prepared by culturing larvae *in vitro* in RPMI 1640 medium (GE Healthcare Life Sciences, South Logan, UT, USA) supplemented with 1× penicillin/streptomycin (Biowest SAS, Nuaillé, France), and 4.5 g/L glucose for 6 days. The culture medium was collected every 24 h and replaced with new medium. Medium collected at each time point was dialyzed against 1× PBS for 48 h at 4 °C and concentrated using Amicon^®^ Stirred Cells (Merck Millipore, Darmstadt, Germany). The quality of protein at each time point was examined by 12% SDS-PAGE. The ESPs exhibiting similar patterns as those observed at 24 h, without degradation, were pooled and subjected to a Coomassie Plus^TM^ Protein Assay (Thermo Fisher Scientific) to determine the protein concentration, followed by storage at −80 °C.

Ten micrograms of ESPs were separated by 12% SDS-PAGE prior to staining with Coomassie Brilliant Blue G250 solution (Bio-Rad, Hercules, CA, USA). Each gel was cut into 16 rectangles, destained with a destaining solution [50 mM NH_4_HCO_3_, 50% (V/V) acetonitrile (ACN)], reduced with 5 mM dithiothreitol (DTT) (GE Healthcare, UK), and alkylated with 250 mM iodoacetamide (IAM; GE Healthcare, UK). The gel pieces were then incubated in the dark for 30 min and dehydrated with 200 mL ACN. The gels were incubated with trypsin (100 ng/mL; Sigma) at 37 °C overnight to digest the proteins to peptides. These peptides were extracted from the gels using 50% (V/V) ACN, dried in a vacuum evaporator (Labconco, Kansas City, MO, USA) and resuspended in 0.1% formic acid. Each peptide suspension was then injected into an Ultimate 3000 nano-liquid chromatography system (Dionex; Surrey, UK) coupled with tandem MS (nanoLC-MS/MS; micrOTOF-Q II, Bruker; Bremen, Germany), and the resulting mass spectra were processed using analysis software (DataAnalysisTM 4.0, Bruker), as described elsewhere [59]. Mascot version 2.4.1 (Matrix Science, London, UK) was used to search the .mgf file against our in-house transcriptome database. Missed cleavage was set to 1. The variable modification setting was cysteine carbamidomethylation and methionine oxidation. Only protein hits from the MASCOT search with ≥95% confidence were reported.

### Two-DE and immunomics

Twenty-five micrograms of ESPs in a 100 μL volume were transferred into a microcentrifuge tube; the interfering substance was removed using a 2-D Clean-Up kit (GE Healthcare), according to the manufacturer’s instructions. The pellet was resuspended in 125 μL of DeStreak rehydration solution containing IPG buffer (0.5% IPG buffer pH 3–10 NL; GE Healthcare) and then transferred into an IPG strip holder; subsequently, an Immobiline DryStrip pH 3–10 NL, 7 cm (GE Healthcare) was placed face down on the solution. After overlaying cover fluid, the strip was allowed to rehydrate in Ettan IPGphor II under the condition of 50 μA/strip and 20 °C for 14 h, and was then placed face up on an Ettan IPGphor Manifold (GE Healthcare). Electrophoresis was performed using the following running conditions: 0.3 kV/h for the initial 30 min, followed by a gradient of 0.3 kV/h for 30 min, 4.0 kV/h for 90 min, and step down and hold at 3.0 kV/h for 35 min. Subsequent steps, including reduction, alkylation, and SDS-PAGE, were performed as described previously [59]. The protein spots were visualized by staining with Coomassie Brilliant Blue G-250 (CBB) dye.

For immunomics, protein spots in the gels were electrically transferred to PVDF membranes (Pall Corporation, Ann Arbor, MI, USA) and allowed to interact with pooled sera from patients with confirmed helminthic infections, including *G. spinigerum*, *Angiostrongylus cantonensis*, cysticercosis, *Strongyloides stercoralis*, sparganosis, and soil-transmitted helminths (STHs) (five patients per pool). Sera from healthy individuals with no history of infection or relevant signs/symptoms were used as negative controls. The reactive patterns were compared, and protein spots specific to *G. spinigerum* infection were identified. These spots were excised from a CBB-stained gel, and the proteins were identified using MS, as described above.

## Results

### Transcriptome assembly from aL3Gs RNA-Seq data

An analysis of aL3Gs transcripts conducted using the Illumina HiSeq platform yielded 61,602,864 raw reads. After removing adaptors, undetermined nucleotides, and low-quality reads, 56,710,418 (92.1%) clean reads were retrieved. The base- and raw read-qualities from NGS sequencing are shown in Supplementary Figure S3 and Supplementary Table S1, respectively. The high-quality reads were then assembled into contigs. The distribution of contig lengths and densities are shown in Supplementary Figure S4. The clean reads were successfully assembled, which generated 117,204 contigs ranging in size between 201 and 22,386 nucleotides (nt; average = 1200 nt) (Table 1, Supplementary Table S1). The contigs were then subjected to protein-coding prediction (Supplementary Table S2 and S3), which yielded 37,099 contigs that could potentially be translated into proteins (Supplementary Table S1). The assembled contigs have been deposited in the Transcriptome Shotgun Assembly (TSA) database under accession number GHIZ00000000.

**Table 1:** Statistical summary of the *de novo* assembled sequences from advanced third-stage *Gnathostoma spinigerum* larvae (aL3Gs).

Parameter	Number
Sequencing results	
Total raw reads	61,602,864
Total clean reads	56,710,418 (92.1%)
*De novo* assembly results	
Total number of contigs	117,204
Average contig length (min-max length)	1,200 nt (201–22,386 nt)
Total number of splice variant groups	86,312
Average splice variant per groups (min-max)	2.2 (1–21)
Annotations results	
CDD annotation (%)	35,850 (96.6%)
UniProt annotation (%)	14,064 (37.9%)
GO annotation (%)	26,259 (70.8%)
Total annotation (%)	37,099 (100.0%)
With homologues in *G. spinigerum*	
Bilateria (%)	7,420 (20.0%)
*Toxocara canis* (%)	5,980 (16.1%)
*Ascaris suum* (%)	5,238 (14.1%)
*Anisakis simplex* (%)	1,674 (4.5%)
*Ascaris lumbricoides* (%)	1,399 (3.7%)
*Enterobius vermicularis* (%)	986 (2.7%)
GO function classification (%)	26,259 (70.8%)
Biological process	11,439
Cellular component	15,358
Molecular function	19,775

### Functional annotation of aL3Gs transcriptomes

A functional annotation of all assembled contigs demonstrated that 35,850 (96.6%) and 14,064 (37.9%) protein-coding transcripts were successfully annotated using the UniProt and NCBI Conserved Domains databases (using BLASTX with an *E*-value cut-off <10^−5^), respectively. Approximately 78.0% of the transcripts had an amino acid identity of more than 40% with orthologs sharing high homology with proteins from nematodes such as *Toxocara canis* (16.1%), *Ascaris suum* (14.1%), and *Anisakis simplex* (5%), respectively (Supplementary Fig. S5). The distribution of the best sequence alignment identities was based on the BLAST *E*-values and is shown in Supplementary Fig. S6. A gene ontology (GO) annotation was used to predict the protein functional group classifications of the aL3Gs transcripts. Here 26,259 (70.8%) of the 37,099 (100%) assembled transcripts could be successfully assigned into at least one of three GO terms encompassing 60 GO assignments. The first group, “Molecular functions,” contained 19,775 transcripts, followed by “Cellular components” and “Biological processes” with 15,358 and 11,439 transcripts, respectively. The predominant terms listed under “Molecular function” were ATP binding (15.0%), nucleic acid binding (7.8%), and metal ion binding (6.5%); the predominant terms listed for “Cellular component” were integral component of membrane (54.6%), nucleus (10.7%), and intracellular (5.5%), while those for “Biological process” were transcription (5.0%), regulation of transcription (4.2%), and metabolic process (4.1%) (Supplementary Fig. S7). After annotation of the transcripts with three different databases, all 37,099 protein-coding contigs could be assigned some possible functions and could have their excretory–secretory potential predicted. Detailed information about the assembled contig annotation is shown in Supplementary Table S1.

### Potential excretory–secretory proteins and functional annotation

Infective-stage aL3Gs cause cutaneous gnathostomiasis, as well as severe visceral gnathostomiasis via infection of the central nervous system and eyes in humans [26]. ESPs released from larvae include several molecules that play important roles in the parasite itself and also host–parasite interaction including nutrient uptake, tissue invasion, migration, inflammation, and immunomodulation [62]. Therefore, the identification and characterization of these proteins could clarify parasite homeostasis and the host–parasite interaction, which facilitate the development of control strategies against gnathostomiasis, including drugs and vaccines. Furthermore, ESPs can be used for the immunodiagnosis of parasitic infection and for patient monitoring after anthelminthic treatment, as previously demonstrated in several studies of parasitic helminth infection [14, 19, 33, 57].

From among 35,850 NCBI CDD-annotated proteins, 171 proteins contained a signal peptide and were categorized as potential classical ESPs, whereas 292 proteins that contained a secretory signal but lacked a signal peptide were categorized as potential non-classical ESPs. Therefore, 463 predicted proteins were identified as potential ESPs of aL3Gs.

**Figure 1:**
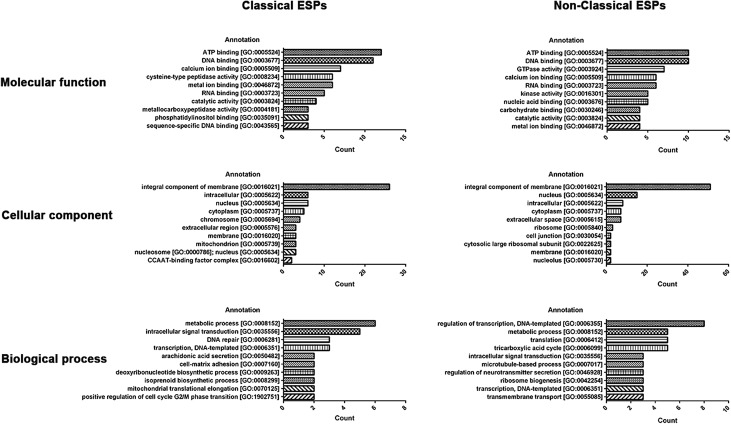
Top 10 enriched gene ontology terms among potential excretory–secretory protein (ESP) transcripts from advanced third-stage larvae of *Gnathostoma spinigerum* (aL3Gs). Classical (left) and non-classical ESPs (right) were categorized into three gene ontology classifications: molecular functions (upper), cellular components (middle), and biological processes (lower). Gene ontology functions are shown on the left *Y*-axis. The number of transcripts is shown on the right *Y*-axis.

The 171 potential classical ESPs were classified into protein functional groups according to GO classification. The largest number of proteins were classified as “Molecular function” (*n* = 106), followed by “Cellular component” (*n* = 77) and “Biological process” (*n* = 64), respectively. ATP binding (*n* = 12) was the most predominant term in “Molecular function,” while integral component of membrane (*n* = 26) was the most predominant term in “Cellular component.” Metabolic process (*n* = 6) was the top GO term identified in “Biological process.” Among the 292 non-classical ESPs, “Molecular function” (*n* = 152) was most abundant, followed by “Cellular component” (*n* = 122) and “Biological process” (*n* = 92). ATP binding (*n* = 10) and DNA binding (*n* = 10) were the most predominant terms in “Molecular function.” For “Cellular component” and “Biological process,” the most predominant terms were integral component of membrane (*n* = 51) and regulation of transcription, DNA-templated (*n* = 8), respectively (Fig. 1).

The top 50 most significant potential functional proteins using BLAST with an *E*-value cut-off < 10^−5^ among both the potential classical and non-classical ESPs are listed in Tables 2 and 3, respectively. Information about all the recruited potential classical and non-classical ESPs is listed in Supplementary Table S4, respectively. The top 50 significant potential classical ESPs included transcripts associated with neuronal proteins (neurexin-4 and sodium-dependent serotonin transporter), cell signaling (diacylglycerol kinase, coiled-coil and c2 domain-containing protein 1-like protein, Raf serine/threonine protein kinase, serine/threonine protein phosphatase, casein kinase II, P-glycoprotein, and phosphatidylethanolamine-binding protein) and proteases (cathepsin B, carboxypeptidase A2, and peptidase M14). Moreover, several transcripts encoding metabolic proteins (5-oxoprolinase, arylsulfatase, and ribonucleoside-diphosphate reductase) and post-translation-related proteins (alpha-1,2-mannosidase, beta-1,3-glucosyltransferase, and 26S protease regulator) were also listed as top 50 significant potential classical ESPs. The top 50 potential non-classical ESPs included transcripts encoding proteins associated with adhesion (integrin alpha pat-2) and cytoskeleton (spectrin beta chain and tubulin beta chain). This category also included groups of transcripts related to metabolism (aconitate hydrolase, phosphoinositide phospholipase, and ectonucleotide pyrophosphatase/phosphodiesterase family member 4), biomolecule synthesis (glycogen synthase and arginyl-tRNA synthetase), and ion transportation (stromal interaction molecule and putative sodium potassium-transporting ATPase subunit beta-3).

**Table 2:** Top 50 significant potential functional proteins in classical excretory–secretory proteins from the advanced third-stage larvae of *Gnathostoma spinigerum* (aL3Gs) transcriptome.

No.	Protein names	Transcript ID	*E*-value
1	Neurexin-4	comp18192_seq0	0
2	Uncharacterized protein	comp6231_seq3	0
3	Uncharacterized protein	comp6345_seq2	0
4	Pecanex-like protein 1	comp6414_seq3	0
5	5-oxoprolinase	comp7397_seq4	0
6	Transcription initiation factor tfiid subunit 2	comp8460_seq2	0
7	Alpha-1,2-Mannosidase (EC 3.2.1.-)	comp960_seq3	0
8	Elongation factor G	comp3740_seq0	1.80E-274
9	Putative diacylglycerol kinase K06A1.6 (DAG kinase)	comp2814_seq4	5.20E-264
10	Uncharacterized protein	comp8944_seq1	9.10E-252
11	Arylsulfatase	comp1959_seq0	7.70E-250
12	Arylsulfatase	comp1959_seq3	1.10E-249
13	26S protease regulatory subunit 7 (Fragment)	comp1510_seq0	9.80E-244
14	26S protease regulatory subunit 7 (Fragment)	comp1510_seq1	9.80E-244
15	Uncharacterized protein	comp5002_seq2	1.00E-217
16	Carboxypeptidase A2	comp6316_seq2	8.20E-214
17	Uncharacterized protein	comp6105_seq2	8.60E-209
18	Carboxypeptidase A2	comp6316_seq0	2.00E-206
19	Elongation factor G	comp3740_seq2	4.70E-187
20	Coiled-coil and c2 domain-containing protein 1-like protein	comp5185_seq6	3.00E-182
21	Coiled-coil and c2 domain-containing protein 1-like protein	comp5185_seq3	3.10E-182
22	Sodium-dependent serotonin transporter	comp7361_seq6	3.50E-173
23	Cathepsin B-like cysteine proteinase 6	comp736_seq0	2.20E-164
24	Cathepsin B-like cysteine proteinase 6	comp736_seq1	2.20E-164
25	Cathepsin B-like cysteine proteinase 6	comp736_seq2	2.70E-164
26	Cathepsin B-like cysteine proteinase 6	comp736_seq4	3.30E-164
27	Cathepsin B-like cysteine proteinase 6	comp736_seq3	3.40E-164
28	Cathepsin B-like cysteine proteinase 6	comp736_seq5	3.40E-164
29	Cre-eff-1 protein	comp16964_seq1	1.70E-163
30	Raf serine/threonine-protein kinase	comp3619_seq2	5.50E-160
31	Cre-eff-1 protein	comp16964_seq0	3.60E-158
32	P-GlycoProtein related	comp22636_seq0	5.00E-157
33	P-GlycoProtein related	comp22636_seq1	5.00E-157
34	Ribonucleoside-diphosphate reductase small chain	comp3298_seq1	2.60E-156
35	Ribonucleoside-diphosphate reductase small chain	comp3298_seq2	2.60E-156
36	Raf serine/threonine-protein kinase	comp3619_seq0	3.10E-146
37	Prolyl 4-hydroxylase subunit alpha-1	comp20829_seq1	2.00E-145
38	Beta-1,3-glucosyltransferase	comp9587_seq1	2.40E-136
39	Uncharacterized protein	comp11306_seq1	1.10E-129
40	Peptidase M14	comp2857_seq0	3.90E-129
41	Uncharacterized protein	comp1752_seq0	1.40E-123
42	Uncharacterized protein	comp1752_seq1	1.40E-123
43	Casein kinase II subunit beta (CK II beta)	comp663_seq4	5.10E-119
44	Uncharacterized protein	comp6326_seq4	4.40E-111
45	Putative RNA 3’-terminal phosphate cyclase-like protein	comp2261_seq3	2.10E-109
46	Flap endonuclease 1 (FEN-1)	comp5588_seq0	5.00E-103
47	Serine/threonine protein phosphatase	comp4127_seq0	3.30E-98
48	Phosphatidylethanolamine-binding protein	comp456_seq1	9.30E-87
49	Uncharacterized protein	comp1050_seq1	1.40E-85
50	Uncharacterized protein	comp9949_seq0	4.10E-84

**Table 3:** Top 50 significant potential functional proteins in non-classical excretory–secretory proteins from the advanced third-stage larvae of *Gnathostoma spinigerum* (aL3Gs) transcriptome.

No.	Protein names	Transcript ID	*E*-value
1	Integrin alpha pat-2	comp1772_seq0	0
2	Glycogen [starch] synthase	comp3052_seq0	0
3	JNK-interacting protein, variant	comp3890_seq1	0
4	Uncharacterized protein	comp3952_seq13	0
5	Uncharacterized protein	comp3952_seq8	0
6	Cytoplasmic FMR1-interacting-like protein	comp5249_seq0	0
7	ATP-dependent RNA helicase DDX46	comp5447_seq2	0
8	Spectrin beta chain	comp899_seq1	0
9	Arginyl-tRNA synthetase (Fragment)	comp3718_seq0	2.80E-289
10	Eukaryotic translation initiation factor 3 subunit D (eIF3d)	comp1927_seq1	4.80E-270
11	Uncharacterized protein	comp6944_seq0	3.30E-263
12	Putative aconitate hydratase 1	comp7480_seq1	1.00E-257
13	Phosphoinositide phospholipase C	comp6826_seq0	1.10E-245
14	BMA-SMG-6, isoform d	comp8286_seq0	5.90E-245
15	Putative aconitate hydratase 1	comp7480_seq4	3.10E-244
16	Uncharacterized protein	comp7268_seq2	9.60E-242
17	Phosphatidylinositide phosphatase SAC2	comp1296_seq9	1.20E-239
18	Tubulin beta chain	comp10855_seq0	1.00E-234
19	Tubulin beta chain	comp10855_seq1	1.00E-234
20	UPF0577 protein-like protein	comp27596_seq0	2.70E-225
21	Tyrosine-protein phosphatase non-receptor type II	comp5184_seq2	2.20E-215
22	Uncharacterized protein	comp4857_seq0	1.70E-214
23	Uncharacterized protein	comp4857_seq1	2.80E-212
24	Uncharacterized protein	comp5818_seq0	1.10E-201
25	Telomerase-binding protein EST1A	comp8286_seq1	6.60E-182
26	Uncharacterized protein	comp7823_seq1	6.00E-173
27	Uncharacterized protein	comp9114_seq3	3.60E-169
28	Histone acetyltransferase	comp2414_seq3	2.70E-166
29	Histone acetyltransferase	comp2414_seq4	2.70E-166
30	Synaptonemal complex protein SC65	comp7780_seq0	3.30E-164
31	Uncharacterized protein	comp2379_seq4	9.20E-164
32	Uncharacterized protein	comp856_seq0	2.80E-162
33	Uncharacterized protein	comp3356_seq0	3.70E-158
34	Uncharacterized protein	comp3172_seq3	3.90E-157
35	Uncharacterized protein	comp2499_seq0	2.70E-154
36	RNA polymerase II elongation factor ELL	comp3620_seq1	2.40E-152
37	Uncharacterized protein	comp7268_seq3	3.80E-147
38	Putative RNA 3’-terminal phosphate cyclase-like protein	comp2261_seq1	7.70E-146
39	Uncharacterized protein	comp856_seq2	8.90E-146
40	Stromal interaction molecule	comp2776_seq0	2.70E-144
41	Galectin	comp442_seq0	1.30E-142
42	Galectin	comp442_seq1	1.30E-142
43	Putative sodium potassium-transporting ATPase subunit beta-3	comp4663_seq0	2.30E-141
44	Nuclear hormone receptor E75	comp5290_seq0	6.80E-138
45	Nuclear hormone receptor E75	comp5290_seq1	6.80E-138
46	Ancient ubiquitous protein 1-like protein	comp1622_seq3	3.90E-136
47	Uncharacterized protein	comp5136_seq2	4.10E-134
48	Ectonucleotide pyrophosphatase/phosphodiesterase family member 4	comp328_seq2	1.20E-131
49	Uncharacterized protein	comp510_seq2	1.40E-130
50	ATP-dependent RNA helicase DDX56	comp4028_seq4	6.00E-127

### Identification of aL3Gs secretory proteins

**Figure 2:**
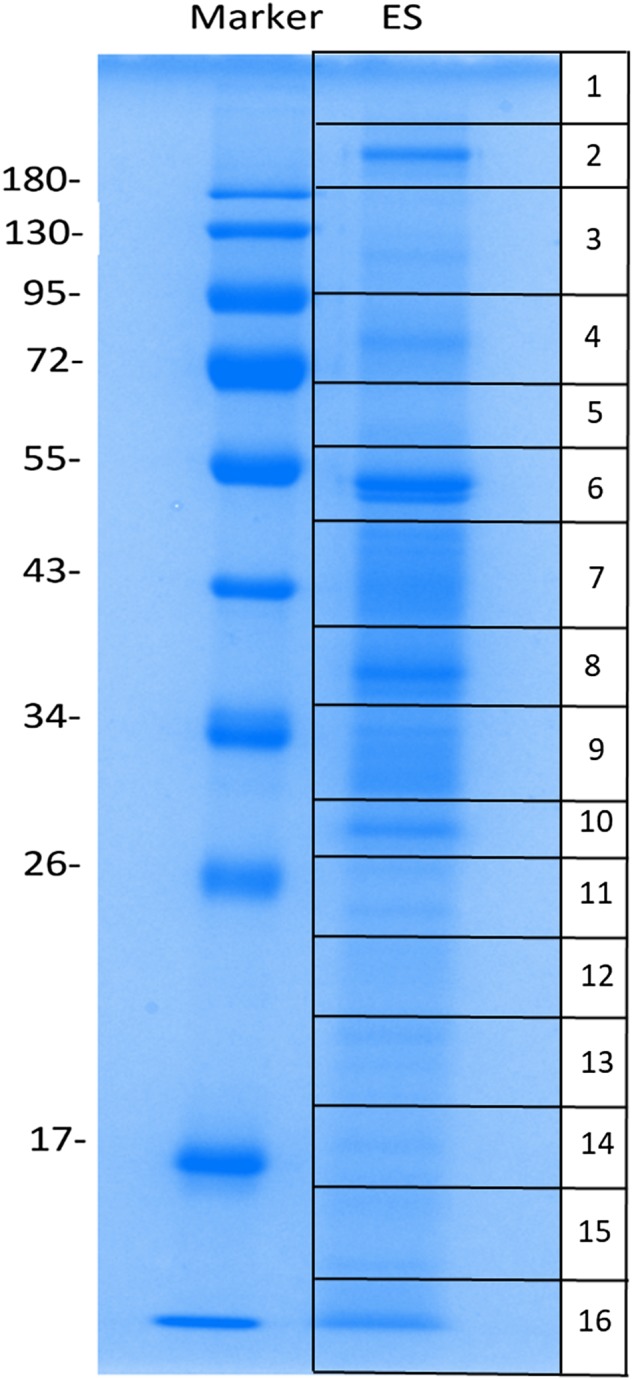
Excretory–secretory proteins from advanced third-stage larvae of *Gnathostoma spinigerum* (aL3Gs). These proteins were examined after separation by one-dimensional gel electrophoresis and excision of whole lanes into 16 rectangular pieces.

ESPs biochemically isolated from aL3Gs were electrically separated by 12% SDS-PAGE, and the proteins were identified using mass spectrometry (MS). The ESPs were distributed at the molecular size range from 10 to 250 kDa. The gel was subsequently cut into 16 equal-sized rectangles prior to MS analysis (Fig. 2). After MS analysis, 29 proteins were identified and allocated into nine functional groups (Table 4). A metalloendopeptidase and serine carboxypeptidase belonging to the protease group were identified as ESPs of aL3Gs. Moreover, the serine protease inhibitor, serpin, was identified as a protease inhibitor group member. Interestingly, several proteins involved in signaling transduction, transcriptional regulation, transportation, and programmed cell death were identified among the ES products of aL3Gs. However, the functions of 19 hypothetical proteins remained unknown and will require further investigation.

**Table 4:** Proteins identified from excretory–secretory products of the advanced third-stage larvae of *Gnathostoma spinigerum* (aL3Gs).

No.	Protein	Transcripts	Score	Molecular weight	% Coverage
Protease					
1	Carboxypeptidase (EC 3.4.16.-)	comp3365_seq0	155	59560	7.1
2	Carboxypeptidase (EC 3.4.16.-)	comp610_seq0	62	124018	2.2
3	Metalloendopeptidase (EC 3.4.24.-)	comp37_seq0	250	93544	8.4
4	Metalloendopeptidase (EC 3.4.24.-)	comp37_seq2	147	101515	4
Unknown					
5	Uncharacterized protein	comp455_seq9	194	158997	3.9
6	Uncharacterized protein	comp455_seq10	184	196865	2.6
7	Uncharacterized protein	comp455_seq11	183	158453	3.2
8	Uncharacterized protein	comp4307_seq0	133	42986	7.7
9	Uncharacterized protein	comp871_seq11	96	454446	0.7
10	Uncharacterized protein	comp1222_seq0	56	72917	2.9
11	Putative uncharacterized protein	comp109_seq0	87	48242	5
12	Uncharacterized protein	comp2379_seq0	50	318310	1.3
13	Uncharacterized protein	comp9_seq4	802	130306	16
14	Uncharacterized protein	comp72_seq1	124	325233	2.4
15	Protein CBG11500	comp10_seq4	62	292750	0.9
16	Uncharacterized protein	comp4152_seq1	47	199737	0.9
17	Uncharacterized protein	comp22_seq2	178	411932	1.7
18	Uncharacterized protein	comp0_seq5	55	456002	0.9
19	Uncharacterized protein	comp23_seq0	341	19720	42.1
Cytoskeletal protein					
19	Talin-1	comp783_seq0	47	413351	1.1
20	Apx/Shroom domain ASD1	comp171_seq0	61	49558	4.8
Protease inhibitor					
21	Serpin-like protein	comp86_seq0	210	68930	4.7
22	Serpin-like protein	comp86_seq1	205	68971	10.6
23	Serpin-like protein (Fragment)	comp86_seq2	63	44673	5.5
Lipid metabolism					
24	MACS_like_1	comp115_seq0	57	64895	1.9
Carbohydrate transport and metabolism					
25	Polypeptide N-acetylgalactosaminyltransferase (EC 2.4.1.-)	comp9_seq1	108	320030	1.4
26	Polypeptide N-acetylgalactosaminyltransferase (EC 2.4.1.-)	comp9_seq2	129	308383	2.2
Transcriptional regulation					
27	Tyrosyl-DNA phosphodiesterase	comp140_seq1	50	87509	1.8
Signaling pathway					
28	PAS domain-containing serine/threonine-protein kinase	comp45_seq2	72	162264	3.5
Catalytic activity					
29	3-isopropylmalate dehydratase large subunit	comp13021_seq0	49	35761	6.7

### Determination of immunodiagnostic target using 2-DE and immunomics

**Figure 3:**
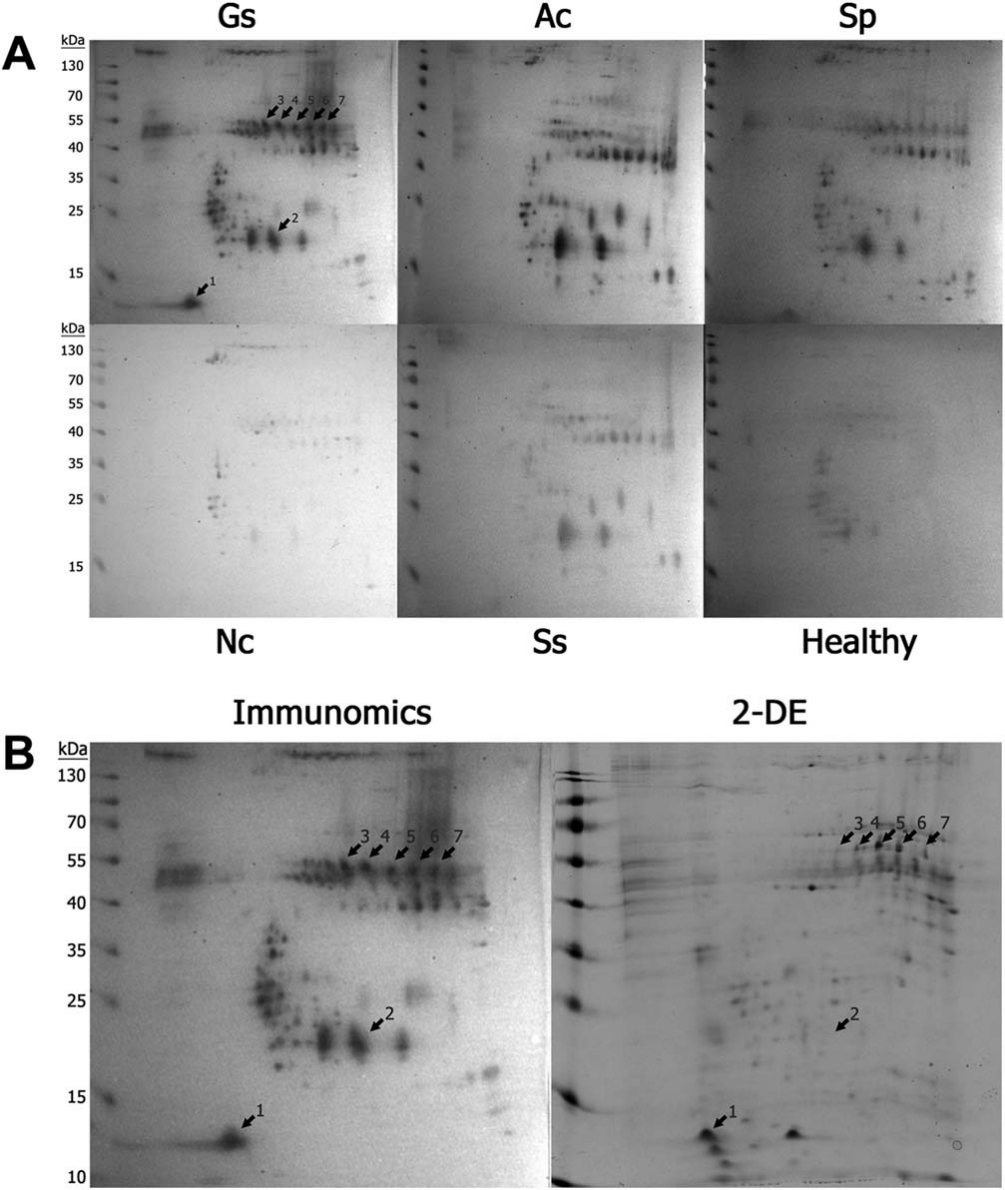
Two-dimensional (2D)-immunomics of advanced third-stage larvae of *Gnathostoma spinigerum* (aL3Gs) reacted with human sera. Two-dimensional (2D)-immunomics of advanced third-stage larvae of *Gnathostoma spinigerum* (aL3Gs) reacted with human sera from patients infected with *G. spinigerum* and other related helminths (A) Gs, *G. spinigerum*-infected human sera; Ac, *Angiostrongylus cantonensis*-infected sera; Sp, sparganosis sera; Nc, neurocysticercosis sera; Ss, *Strongyloides stercoralis*-infected sera; Healthy, healthy sera. After comparing the patterns of reactivity among the serum samples, seven spots were found to react specifically with *G. spinigerum*-infected sera. The spot patterns were compared with the 2D-gel, from which the proteins were excised for identification using mass spectrometry (B). Immunomics, 2D-immunomics; 2DE, 2D-gel electrophoresis. Arrows indicate the spots that reacted specifically with *G. spinigerum*-infected human sera.

Western blot analysis for the detection of IgG antibodies against the 24-kDa CWA has been used as a gold standard method for diagnosing *G. spinigerum* infection. However, the identification of additional targets could improve the reliability of gnathostomiasis immunodiagnosis. Accordingly, we conducted a 2-DE/immunomic analysis to determine potential targets for immunodiagnosis. A comparison of the reactive patterns of pooled human serum samples from patients with selected helminthic infections was performed, and the result demonstrated that seven spots were specifically recognized by pooled sera from patients with *G. spinigerum* (Fig. 3). The spots were collected and subjected to MS to assess the protein types; Table 5 includes a list of these proteins. Several spots (spots 4–7) predominantly recognized by *G. spinigerum*–human pooled sera yielded proteins with a molecular weight of 48 kDa but different pI values and were identified as serine protease inhibitors (serpins; GsSerp). The nucleotide and deduced amino acid sequences of GsSerp (comp9_seq4) were obtained from the aL3Gs transcriptomic database and used for further bioinformatics analysis.

**Table 5:** Immunogens identified from excretory–secretory products of advanced third-stage larvae of *Gnathostoma spinigerum* (aL3Gs).

Spot no.	Protein no.	Transcripts	Protein	Score	Molecular weight	% coverage	Sequence
1	1	comp5644_seq4	Serine_rich_NEDD9	94	74822	2.7	CTVSPLR
							YFLLSQLFICTK
2	1	comp7822_seq0	Catalase heme-binding enzyme	96	101056	2.1	LTDLLDFHS
							ECNLQLSTSK
3	1	comp65414_seq0	Uncharacterized protein	76	13604	5.6	LAVSDMR
	2	comp6066_seq0	Pyrroline-5-carboxylate reductase	72	104867	0.8	RATVLSPR
	4	comp6937_seq0	Phospho-2-dehydro-3-deoxyheptonate aldolase	96	113706	1.7	LLLASLNP
							SLFIGGTVA
	5	comp7822_seq0	Catalase heme-binding enzyme	90	102122	2.1	LTDLLDFHS
							ECNLQLSTSK
4	1	comp9_seq4	Serpin	153	130306	4.3	SSPISAIFTSFK
							TATGGFVKGTVEER
							QVISPVAESLALGAVYEGSQDETR
5	1	comp9_seq4	Serpin	930	130306	14.3	WKNPLTK
							MFHAETR
							YNFNKPK
							GSGEYLYK
							TNYEAVEK
							QIGIAMFGTK
							IAATETEEWVK
							SSPISAIFTSFK
							TATGGFVKGTVEER
							FSYEQQFLTSLK
							ESGEFGGKQVSFLK
							FVANRPFFFALVR
							RFSYEQQFLTSLK
							IAATETEEWVKTATGGFVK
							GESYGELEKEINGETIILK
							QVISPVAESLALGAVYEGSQDETR
							QVISPVAESLALGAVYEGSQDETRK
	2	comp23_seq0	Uncharacterized protein	435	19720	36.5	QIGIAMFGTK
							FSYEQQFLTSLK
							RFSYEQQFLTSLK
							LADFGLSLFQLSSQPGK
							QVISPVAESLALGAVYEGSQDETR
							QVISPVAESLALGAVYEGSQDETRK
	3	comp7822_seq0	Catalase heme-binding enzyme	98	102122	2.1	LTDLLDFHS
							ECNLQLSTSK
6	1	comp9_seq4	Serpin	559	130306	9.7	WKNPLTK
							YNFNKPK
							SSPISAIFTSFK
							TATGGFVKGTVEER
							FSYEQQFLTSLK
							FVANRPFFFALVR
							QVISPVAESLALGAVYEGSQDETR
							QVISPVAESLALGAVYEGSQDETRK
							NSTAPHDSTVMKVMAENVLRPHVR
	2	comp23_seq0	Uncharacterized protein	256	19697	30.3	FSYEQQFLTSLK
							LADFGLSLFQLSSQPGK
							QVISPVAESLALGAVYEGSQDETR
							QVISPVAESLALGAVYEGSQDETRK
7	1	comp9_seq4	Serpin	176	130306	4.3	SSPISAIFTSFK
							TATGGFVKGTVEER
							QVISPVAESLALGAVYEGSQDETR
	2	comp7822_seq0	Catalase heme-binding enzyme	118	101056	2.2	QTCSISIR
							LTDLLDFHS
							ECNLQLSTSK
	3	comp22636_seq0	AJAP1/PANP C-terminus	97	53020	5.3	ATTSVMAVSIPR
							KLTVVSIECIMIR

### Serine protease inhibitor of *G. spinigerum*

**Figure 4:**
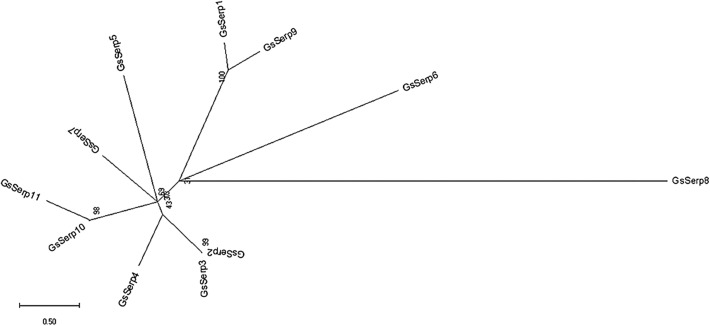
An unrooted maximum likelihood tree showing the genetic relationships among the GsSerp isoforms. The GsSerp isoforms were identified in advanced third-stage larvae of *Gnathostoma spinigerum* (aL3Gs). The nucleotide and amino acid sequences of GsSerp isoforms were submitted to the NCBI database, and accession numbers are provided in Table 6.

**Figure 5:**
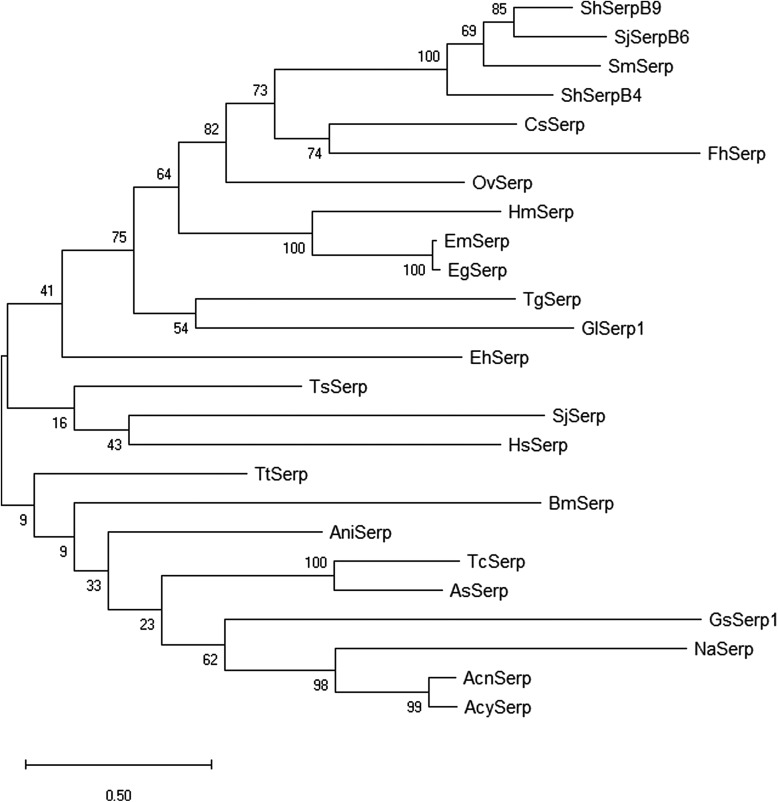
Phylogenetic tree of GsSerp1 and its orthologs. The tree demonstrates the close relationships of GsSerp1 with hookworm and Ascarididae serpins. The accession numbers of GsSerp1 and orthologs included in this analysis are provided in Table 6.

**Figure 6:**
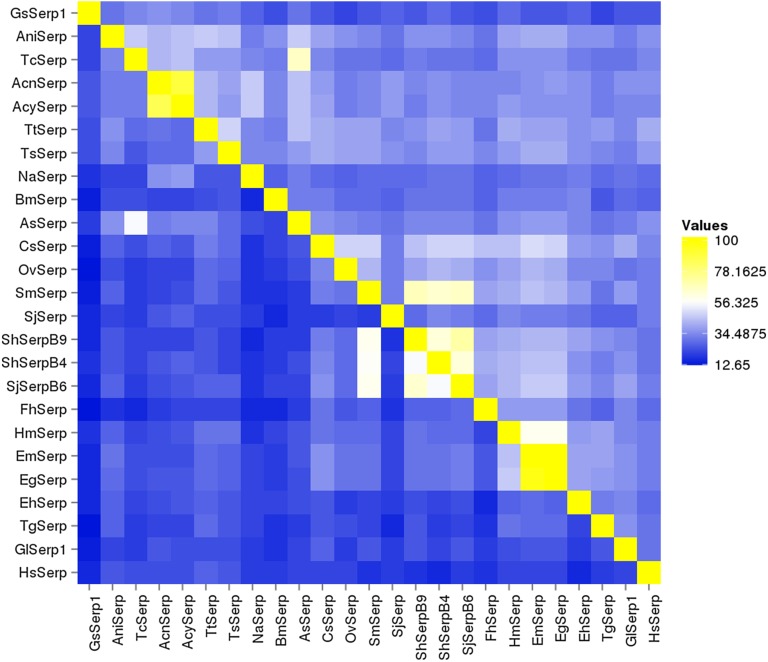
Homology heatmaps. Homology heatmaps for comparisons of the percentage identity (lower left) and similarity (upper right) between GsSerp1 and its orthologs. The percentage identity and similarity values are indicated by different box colors (right).

The nucleotide and amino acid sequences of the identified GsSerp (comp9_seq4) were submitted to the NCBI database for accession number assignment and renamed to GsSerp1. Other GsSerp isoforms were also identified from the transcriptomic database and subjected to accession number assignment by the NCBI database. Eleven different isoforms (GsSerp1-11) were obtained; their accession numbers are provided in Table 6. The amino acid homology and relationships among GsSerp isoforms were analyzed through a multiple alignment conducted using MUSCLE software, after which a phylogenetic tree was constructed based on a maximum likelihood analysis with 1000 bootstrap replications using the MEGA X program [34]. Our results demonstrated that GsSerp1 was most closely related to GsSerp9 but distinctly separated from the other isoforms (Fig. 4). To evaluate the diagnostic potential of GsSerp1, we included it in a homology comparison with serpins of other parasitic helminths, protozoa, and humans (Table 6) using a multiple alignment via MUSCLE software (https://www.ebi.ac.uk/Tools/msa/muscle/) and the MEGA X program [34]. A phylogenetic tree was then constructed using the abovementioned parameters, and the tree demonstrated close relationships of GsSerp1 with both hookworm (*A. caninum*, *A. ceylanicum* and *N. americanus*) and Ascarididae (*A. suum* and *T. canis*) serpins (Figure 5). The pairwise sequence identity and similarity were calculated from a multiple alignment of GsSerp1 and its orthologs via the Sequence Identity and Similarity program (http://imed.med.ucm.es/Tools/sias.html) and were subsequently visualized as a heatmap table using heatmapper software (http://heatmapper.ca). The homology heatmap suggested that GsSerp1 shared low identity (less than 30%) with all orthologs, even hookworm and Ascarididae serpins (Fig. 6).

**Table 6:** Amino acid sequences and the corresponding accession numbers used in this study.

Name of protein	Species	Accession no.
GsSerp1	*Gnathostoma spinigerum*	MK128410
GsSerp2	*G. spinigerum*	MK128411
GsSerp3	*G. spinigerum*	MK128412
GsSerp4	*G. spinigerum*	MK128413
GsSerp5	*G. spinigerum*	MK128414
GsSerp6	*G. spinigerum*	MK128415
GsSerp7	*G. spinigerum*	MK128416
GsSerp8	*G. spinigerum*	MK128417
GsSerp9	*G. spinigerum*	MK128418
GsSerp10	*G. spinigerum*	MK128419
GsSerp11	*G. spinigerum*	MK128420
AniSerp	*Anisakis simplex*	CBX25525.1
TcSerp	*Toxocara canis*	KHN72316.1
AcnSerp	*Ancylostoma caninum*	RCN49152.1
AcySerp	*A. ceylanicum*	EPB78059.1
TtSerp	*Trichuris trichiura*	CDW55271.1
TsSerp	*Trichinella spiralis*	CBX25723.1
NaSerp	*Necator americanus*	ETN83761.1
BmSerp	*Brugia malayi*	AAB65745.1
AsSerp	*Ascaris suum*	AEH42098.1
CsSerp	*Clonorchis sinensis*	ADI60059.1
OvSerp	*Opisthorchis viverrini*	OON15112.1
SmSerp	*Schistosoma mansoni*	CCD60352.1
SjSerp	*S. japonicum*	AAK57435.1
SjSerpB6	*S. japonicum*	CAX69453.1
ShSerpB4	*S. haematobium*	KGB37772.1
ShSerpB9	*S. haematobium*	KGB42280.1
FhSerp	*Fasciola hepatica*	PIS86324.1
HmSerp	*Hymenolepis microstoma*	CDS31921.1
EmSerp	*Echinococcus multilocularis*	CDS35970.1
EgSerp	*E. granulosus*	CDS22753.1
EhSerp	*Entamoeba histolytica*	EAL44876.1
TgSerp	*Toxoplasma gondii*	CEL78311.1
GlSerp1	*Giardia lamblia*	XP_001706503.1
HsSerp	*Homo sapiens*	CAL47031.1

## Discussion

In Southeast Asia, *G. spinigerum* is a major causative agent of gnathostomiasis, a life-threatening parasitic disease in both humans and animals. However, the lack of available genome, transcriptome, and proteome databases remains a hindrance to the development of effective prevention and control strategies, including drugs, vaccines, and diagnostic methods. In this study, a pioneer transcriptomic database of aL3Gs was constructed in a 2 × 150 bp paired-end (PE) configuration with incorporated bioinformatics analysis using the Illumina HiSeq platform. A total of 61,602,864 paired reads were obtained and subjected to a quality assessment with a quality score (value of Q30) greater than 80%. In addition, 89.6% of raw reads could be aligned to assembled sequences, indicating the presence of high-quality sequences and, consequently, an effective assembly and downstream data analysis was performed. The resulting contigs were arranged in groups, which enabled us to obtain transcript variants. We identified 86,312 splice variant groups (mean = 2.18 splice variants per group), suggesting a broad transcript diversity in this sequence database.

A *G. spinigerum* genome is not currently available [38]; therefore, a *de novo* assembly was obtained in this study to generate assembled sequences. High-quality sequences were subjected to assembly using the Bridger [11] assembler, which was selected because it provided the best accuracy while maximizing the utility of the PE reads. Bridger also enables the most complete reconstruction of transcripts, compared with other assemblers [50].

Gene ontology annotation demonstrated that transcription processes were highly represented within the biological process category of aL3Gs, and the result indicated the expression of several genes responsible for transcription factors and transcription regulation, including the transcription elongation factor 1 homolog, transcription elongation factor SPT5, RuvB-like helicase, general transcription factor IIH subunit 1, histone deacetylase, histone acetyltransferase, repressor of RNA polymerase III transcription MAF1, Zinc finger homeobox protein 3, and DNA-directed RNA polymerase subunit. The expressions of these genes suggest a high level of transcriptional activity during this infective stage. As aL3Gs is a developmental stage during which the organism transitions between stages, changes in the expression of genes with predicted functions in many biological processes are needed to enable the physical and physiological changes associated with growth and development [37].

Integral membrane proteins, especially transmembrane proteins, were the most frequently identified cellular component among aL3Gs, and included transporter proteins (ABC transporter ATP-binding protein, amino acid transporter, metal transporter cnnm4, organic cation transporter protein, sodium- and chloride-dependent glycine transporter 2, and zinc transporter 1) and ion channel proteins (ligand-gated ion channel, organic cation transporter protein, solute carrier organic anion transporter family member, acid-sensing ion channel 1, TWiK family of potassium channels protein 7, and voltage-dependent calcium channel type A subunit alpha-1). Several studies have reported a response of transporter and ion channel proteins to translocate a substrate across cell membranes. The high levels of expression of various transporter proteins indicate that various nutrients are required for growth and development in this stage [18, 68]. However, this phenomenon remains to be clarified in other developmental stages.

ATP binding activity, especially kinase activity, was the most active function in aL3Gs; specifically, this activity involved the mitogen-activated protein (MAP) kinase family, which includes dual-specificity mitogen-activated protein kinase 4, dual-specificity mitogen-activated protein kinase 6, dual-specificity mitogen-activated protein kinase 7, and dual-specificity mitogen-activated protein kinase mek-2. In mammals, MAP kinases are the major components of pathways that control embryogenesis, cell differentiation, cell proliferation, and cell death [48]. In parasites, however, little is known about protein kinases, although they might play key roles in proliferation, differentiation, and invasion [52]. Several studies conducted in *Plasmodia*, *Leishmania*, and *Toxoplasma* found that MAP kinase signaling events play a role in parasitic immune evasion and thus promote survival in the host [8, 25, 30, 56]. The extrapolation of these findings to *G. spinigerum* implies that MAP kinase signaling pathways are involved in several indispensable activities.

Prediction of potential ESPs of aL3Gs was used as a guide to verify proteins with a potential to excrete inside the parasite or secrete outside to interact with the host environment. However, recently available prediction programs could not differentiate between excretory- and secretory proteins, which need to be intensively proven by experimental evidence, and this will be a priority in future studies. In the “Molecular function” category, ATP binding was the top GO term identified for both potential classical and non-classical ESPs, which included protein kinases (nucleoside diphosphate kinase, Raf serine/threonine protein kinase, pantothenate kinase 1, and 3-phosphoinositide-dependent protein kinase 1) and replication, transcription, and translation (ATP-dependent RNA helicase DDX56, regulator of nonsense transcripts 1, and 26S protease regulatory subunit 7). These transcripts may directly affect growth regulation, development, and survival of the parasite, as mentioned above. Moreover, the secreted forms of these proteins may act as an extracellular signal to regulate the host at a molecular level and thus provide suitable niches for parasite growth and development or the evasion of host defense mechanisms. ESPs of the liver fluke *Opisthorchis viverrini* were shown to stimulate fibroblast cell proliferation by upregulating genes that encoding proteins associated with metabolism, signal transduction, replication, transcription and translation, matrix and structural proteins, and cell cycle [60, 61]. ESPs derived from *Heligmosomoides polygyrus* could stimulate host regulatory responses by inducing Foxp3^+^ Treg cells and the TGF-β signaling pathway [24]. Regarding *G. spinigerum*, ESPs of aL3Gs impaired the human monocyte phagocytosis by downregulating FcγRI expression [6].

Regarding the cellular component, most potential classical and non-classical ESPs transcripts were categorized into the integral component of membrane category. Transportation-related proteins were represented, including sodium-dependent serotonin transporter, organic solute transporter alpha-like protein, putative transporter, and heme transporter hrg-1. In *Schistosoma mansoni*, a serotonin transporter facilitated the distribution of serotonin into neurons or adjacent glial cells to contribute to parasite movement and muscle contraction. Moreover, the serotonin transporter transported exogenous serotonin, which may have affected the uptake of host-derived serotonin [47]. However, the role of host serotonin on parasite biochemical physiology has not yet been addressed and requires further investigation. Regarding biological processes, the metabolic process and regulation of transcription, DNA templates were the most enriched terms for classical and non-classical ESPs, respectively. Alpha-1,2-mannosidase, a classical ESP, is essential for the synthesis and processing of N-linked glycoproteins [27]. In skin-grafted mice, alpha-1,2-mannosidase was strongly expressed in alloantigen-reactive Treg cells that suppressed allograft rejection [39]. However, the role of exogenous alpha-1,2-mannosidase, especially when derived from helminths, in Treg stimulation has not been determined and should be explored.

The top 50 significant transcripts of potential classical ESPs from aL3Gs included a significant proportion of metalloproteases. This result was consistent with our proteomic analysis of aL3Gs-ESPs, which identified metalloproteases as a predominant protease. In a previous study, metalloproteases secreted from the aL3 of *G. binucleatum* could degrade gelatin, fibronectin, and antibodies and thus might contribute to parasitic tissue invasion, migration, and immune evasion [64]. A 24-kDa protein identified as a matrix metalloprotease has been used for the immunodiagnosis of human gnathostomiasis [29, 63]. Furthermore, proteins involved in carbohydrate and lipid metabolism comprised a significant proportion of the top 50 significant transcripts of non-classical ESPs, as well as the secretomic results. These findings indicate that these proteins not only have roles in the regulation of parasite metabolism but may also be necessary to prepare a microenvironment suitable for growth and colonization in the host. In *S. japonicum*-infected mice, significant changes were observed in uracil, lipid, and carbohydrate metabolism in association with disease progression and the worm burden [66]. However, both the predicted ESPs and secretomic data included large numbers of proteins with unknown functions (i.e., uncharacterized proteins), which may be a consequence of the lack of a genome database and the inability to clarify these transcripts and achieve reliable annotation. Therefore, a *G. spinigerum* genome project will be set as a research priority as soon as possible.

In 2D-immunomics of aL3Gs-ES, seven protein spots were specifically recognized by *G. spinigerum*-infected human sera, and MS identified the serine protease inhibitor serpin in several spots. Serpins are classified into inhibitor family I4, the largest and most diverse superfamily of protease inhibitors; members of this family have been identified in viruses, microorganisms, plants, and animals [12, 51]. Although serpins mainly inhibit serine proteases, some members can inhibit caspases and papain-liked cysteine proteases. Moreover, some serpins exhibit non-inhibitory functions such as molecular chaperone activity, hormone transportation, or even tumor suppression [36]. Regarding immunodiagnosis, *T. spiralis* serpin exhibited promise as an antigen for the immunodiagnosis of swine trichinosis, and yielded 100% rates for both sensitivity and specificity [45]. Furthermore, recombinant serpin derived from *S. mansoni* was shown to differentially diagnose *S. mansoni* infection from other related diseases and *S. haematobium* infection [58]. By contrast, the diagnostic potential of serpin from *G. spinigerum* (GsSerp) remains unknown, due to a lack of previous identification and characterization. In the aL3Gs transcriptomic database presented herein, 11 GsSerp isoforms were identified and their relationship was analyzed using a phylogenetic tree. The wide range of homologies and categorization of these isoforms into different clades might suggest that these proteins perform multiple functions required for parasite biology, including growth, development, mating, fertilization, and immune evasion. The SjSerp isoforms identified from each developmental stage of *S. japonicum* preferably inhibited different serine proteases; for example, SjB10 inhibited pancreatic elastase [42], whereas a novel isoform, SjSPI, inhibited chymotrypsin, trypsin, and thrombin [67]. In *S. mansoni*, SmSprQ did not inhibit host serine proteases but regulated the homeostasis of cercarial elastase [49].

To predict the immunodiagnostic potential of GsSerp, the amino acid sequence of GsSerp1 was determined following its specific detection by *G. spinigerum*-infected human sera. Subsequently, a multiple alignment and pairwise sequence homology were analyzed to calculate the percent identity and percent similarity of GsSerp1 with the orthologs. The low sequence identity (less than 30%) between GsSerp1 and its orthologs indicates that this *G. spinigerum* antigen is unique and may lead to the development of a reliable immunodiagnostic method. Although serpins derived from other *Gnathostoma* spp. should be subjected to an analysis, these are not currently available. Future studies will address the production of recombinant GsSerp1 and evaluate immunodiagnostic potential, thus clarifying the capability and reliability of GsSerp1 in this context.

## Conclusions

The transcriptomic database of aL3Gs constructed in this study led to an understanding of basic parasitic molecular biology and the identification of multiple transcripts that may be associated with parasite growth, development, and survival. The predicted ESPs, together with secretomic analysis, have highlighted key biological, physiological, and immunomodulatory molecules that may be indispensable to the parasite life cycle. A 2D-immunomic analysis of aL3Gs-ES identified the serpin GsSerp1 as a promising candidate for the further development of immunodiagnostic protocols. Moreover, the output from an integrated transcriptomics and secretomic analysis not only appears to enhance our understanding of parasite biology, but also facilitates the discovery of novel drugs and vaccines to respectively treat and prevent this harmful type of helminthiasis.

## List of abbreviations

2DE: 2D-gel electrophoresis; aL3Gs: advanced third stage larva of *G. spinigerum*; CDD: Conserved Domains Database; CDS: Coding sequence; CWA: crude worm antigen; ESPs: excretory–secretory proteins; GO: Gene ontology; ML: maximum likelihood analysis; MS: mass spectrometry; NGS: Next-generation sequencing; ORFs: Open reading frames; PE: paired-end; Serpin: serine protease inhibitor; SIAS: Sequence identity and similarity program; SRA: Sequence read archive; UniProt: The universal protein knowledgebase.

## Ethics approval and consent to participate

Helminth-infected human sera and healthy sera used in this study were provided by the Immunodiagnosis for Helminthiasis Unit, Department of Helminthology, Faculty of Tropical Medicine, Mahidol University, under the approval of the Ethics Committee of the Faculty of Tropical Medicine, Mahidol University (no. MUTM 2011-050-01).

## Availability of data and materials

RNA-Seq reads have been deposited in the NCBI-Sequence Read Archive (SRA) database under accession number SRR8137628. This Transcriptome Shotgun Assembly project has been deposited in DDBJ/EMBL/GenBank under the accession number GHIZ00000000. The version described in this paper is the first version, GHIZ01000000. Mass spectrophotometric data were submitted to the PRIDE database. Data are available via ProteomeXchange with identifier PXD013551.

## Competing interests

The authors declare that they have no competing interests.

## Authors’ contributions

SN and PAD conceived and designed the study. SN, OR, OP, PM and PAD performed the experiments. SN, OR, OP, PC and PAD analyzed the data. SN, OR, OP and PAD drafted the manuscript. PC performed bioinformatics analysis and data management. PD supervised study design, study implementation and manuscript revision. All authors read and approved the final manuscript.

## Supplementary Material

Supplementary material is available at https://www.parasite-journal.org/10.1051/parasite/2019033/olm
Supplementary Tables *Supplementary Table S1*: Statistical summary of raw read and detailed information of assembled transcript BLAST annotation (XLSX 9.7 MB).
*Supplementary Table S2*: Predicted peptide sequences (XLSX 10 MB).
*Supplementary Table S3*: Predicted peptide sequences (cont.) (XLSX 10 MB).
*Supplementary Table S4*: Potential functional proteins among classical and non-classical excretory–secretory proteins from the advanced third-stage larvae of *Gnathostoma spinigerum* (aL3Gs) transcriptome (XLSX 290 KB). *Supplementary Figure S1*: The quality and quantity of the total RNA sample (PDF 522 KB). *Supplementary Figure S2*: >Bioinformatics workflow of the excretory–secretory proteins (PDF 110 KB). *Supplementary Figure S3*: Base quality distribution of the read lengths (PDF 849 KB). *Supplementary Figure S4*: Density (smoothed frequency) of the contigs according to the length of the advanced third-stage larvae of the *Gnathostoma spinigerum* (aL3Gs) data set (PDF 145 KB). *Supplementary Figure S5*: Species distribution of BLAST hits against the UniProt database (PDF 212 KB). *Supplementary Figure S6*: *E-*value of the identity distribution (PDF 152 KB). *Supplementary Figure S7*: Top 20 enriched gene ontology (GO) terms among advanced third-stage larvae of *Gnathostoma spinigerum* (aL3Gs) transcripts (PDF 233 KB).
