# Supplementary material for: Transcriptome and excretory–secretory proteome of infective-stage larvae of the nematode Gnathostoma spinigerum reveal potential immunodiagnostic targets for development
Source: Parasite. 2019 Jun 5;26:34. doi: 10.1051/parasite/2019033 (PMC6550564; doi:10.1051/parasite/2019033)
Supplement: Supplementary file 2 — Supplementary Figure S1: The quality and quantity of the total RNA sample (PDF 522 KB). [file parasite-26-34-s5.pdf]

Supplementary Figure S1

The quality and quantity of the total RNA sample

Assay Class: Eukaryote Total RNA Nano
Data Path: C:\...Eukaryote Total RNA Nano_DE24802309_2017-06-15_11-26-19.xad

Created: 6/15/2017 11:26:19 AM
Modified: 6/15/2017 2:26:48 PM

Electropherogram Summary

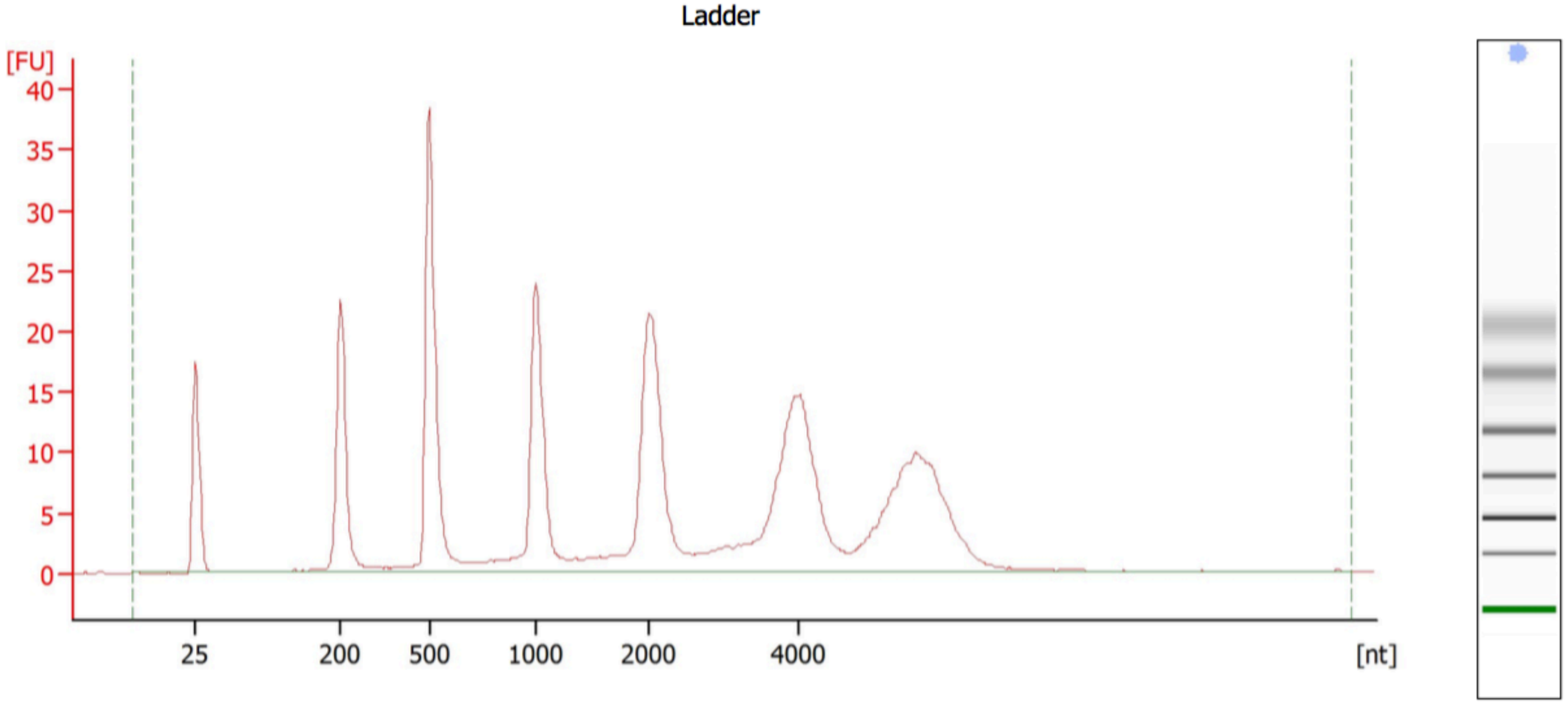

Overall Results for Ladder

RNA Area:	330.7	Result Flagging Color:	
RNA Concentration:	150 ng/µl	Result Flagging Label:	All Other Samples

Assay Class:	Eukaryote Total RNA Nano	Created:	6/15/2017 11:26:19 AM
Data Path:	C:\...Eukaryote Total RNA Nano_DE24802309_2017-06-15_11-26-19.xad	Modified:	6/15/2017 2:26:48 PM

Electropherogram Summary Continued ...

GsL3-PA [3XDilution]

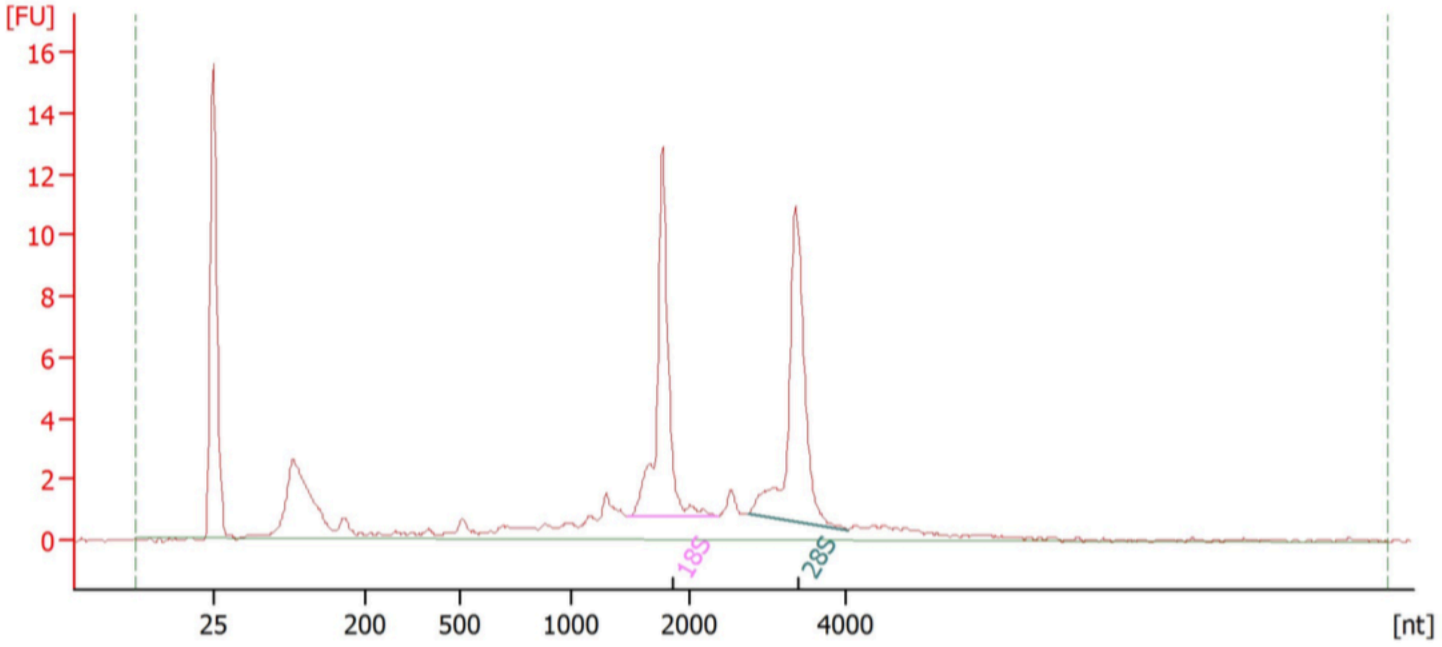

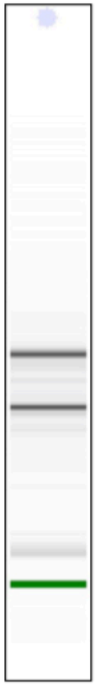

Overall Results for sample 7 : GsL3-PA

RNA Area:	76.5	RNA Integrity Number (RIN):	8.9 (B.02.08)
RNA Concentration:	35 ng/µl	Result Flagging Color:	
rRNA Ratio [28s / 18s]:	1.1	Result Flagging Label:	RIN: 8.90

Fragment table for sample 7 : GsL3-PA

Name	Start Size [nt]	End Size [nt]	Area	% of total Area
18S	1,485	2,349	15.7	20.5
28S	2,737	4,014	16.7	21.8
