# Supplementary figures and images for: Transcriptome and excretory–secretory proteome of infective-stage larvae of the nematode Gnathostoma spinigerum reveal potential immunodiagnostic targets for development
Source: Parasite. 2019 Jun 5;26:34. doi: 10.1051/parasite/2019033 (PMC6550564; doi:10.1051/parasite/2019033)

# **Supplementary Figure S2**

Bioinformatics workflow of the  
excretory–secretory proteins

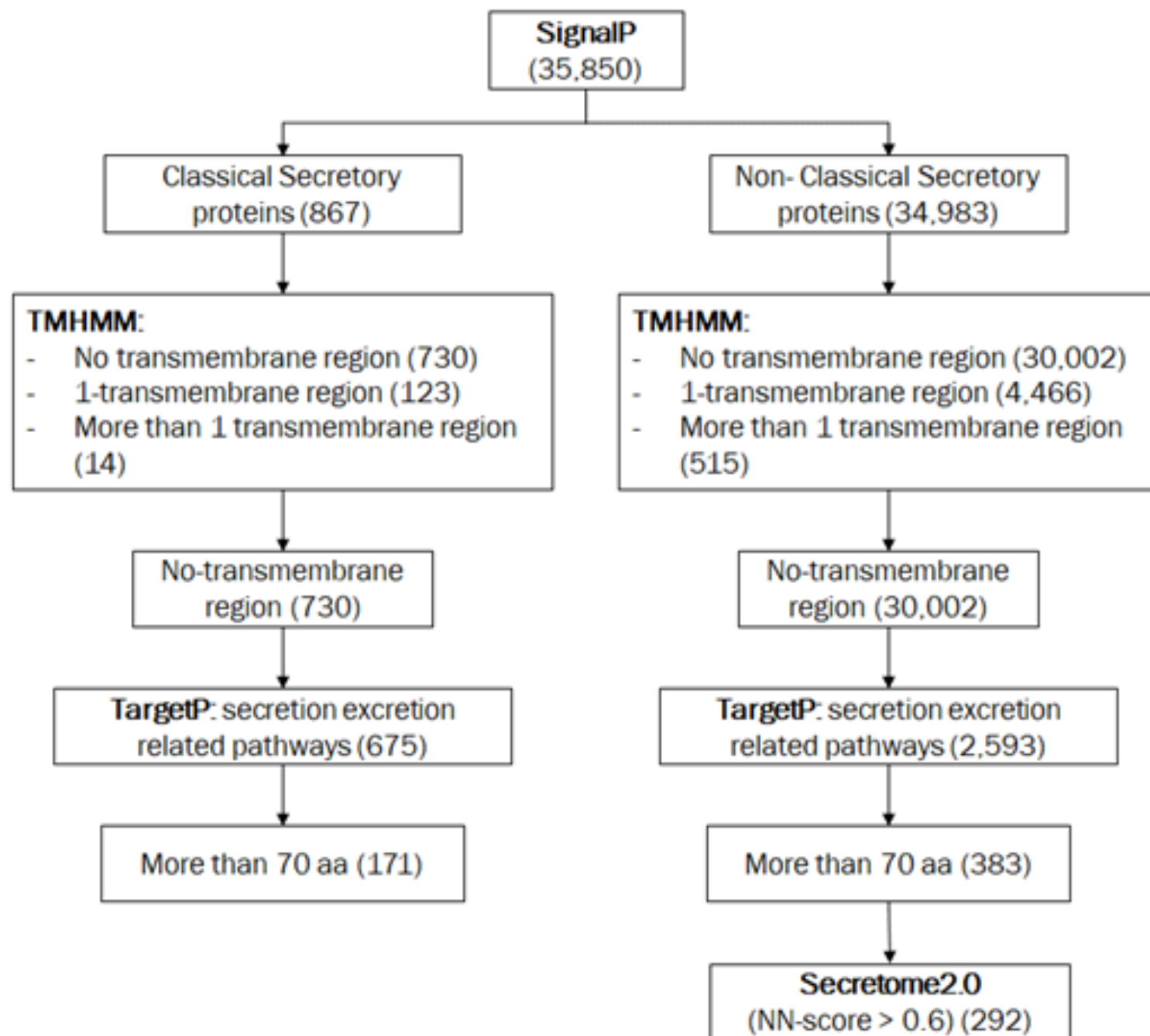

Supplement: Supplementary file 3 — Supplementary Figure S2: >Bioinformatics workflow of the excretory–secretory proteins (PDF 110 KB). [file parasite-26-34-s6.pdf]

## **Supplementary Figure S6**

*E*-value of the identity distribution

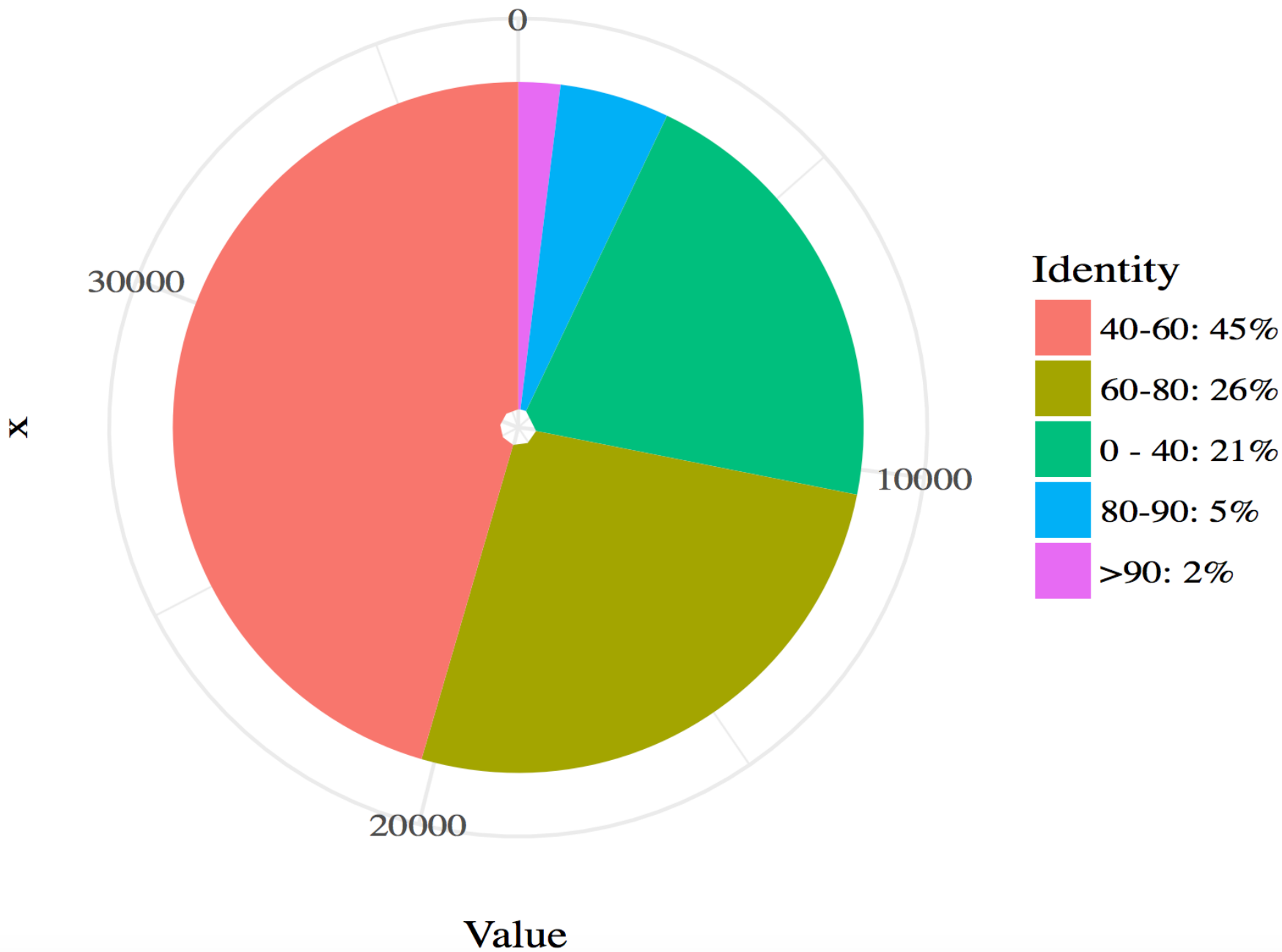

Supplement: Supplementary file 7 — Supplementary Figure S6: E-value of the identity distribution (PDF 152 KB). [file parasite-26-34-s10.pdf]
