# Supplementary material for: Transcriptome and excretory–secretory proteome of infective-stage larvae of the nematode Gnathostoma spinigerum reveal potential immunodiagnostic targets for development
Source: Parasite. 2019 Jun 5;26:34. doi: 10.1051/parasite/2019033 (PMC6550564; doi:10.1051/parasite/2019033)
Supplement: Supplementary file 4 — Supplementary Figure S3: Base quality distribution of the read lengths (PDF 849 KB). [file parasite-26-34-s7.pdf]

# Supplementary Figure S3

Base quality distribution of the read lengths

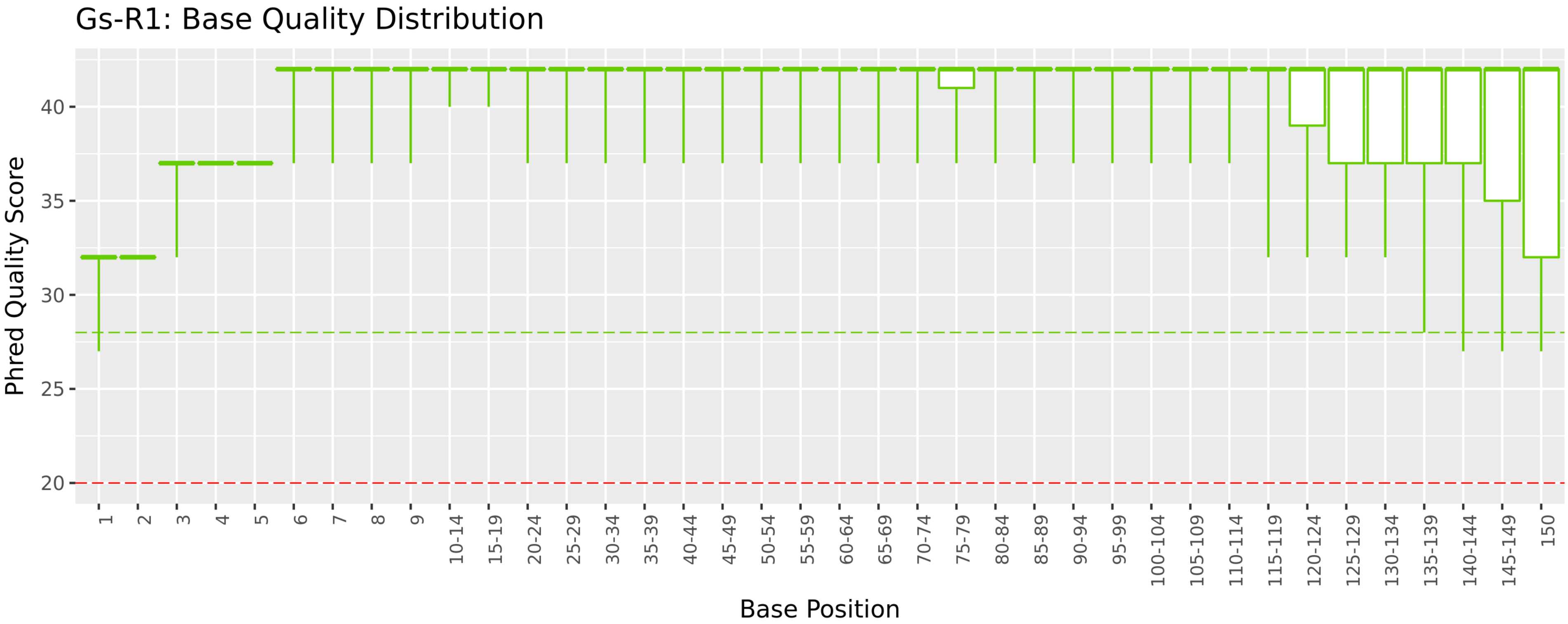

Gs-R1: Base Quality Distribution
Phred Quality Score
40
35
30
25
20
1
2
3
4
5
6
7
8
9
10-14
15-19
20-24
25-29
30-34
35-39
40-44
45-49
50-54
55-59
60-64
65-69
70-74
75-79
80-84
85-89
90-94
95-99
100-104
105-109
110-114
115-119
120-124
125-129
130-134
135-139
140-144
145-149
150
Base Position

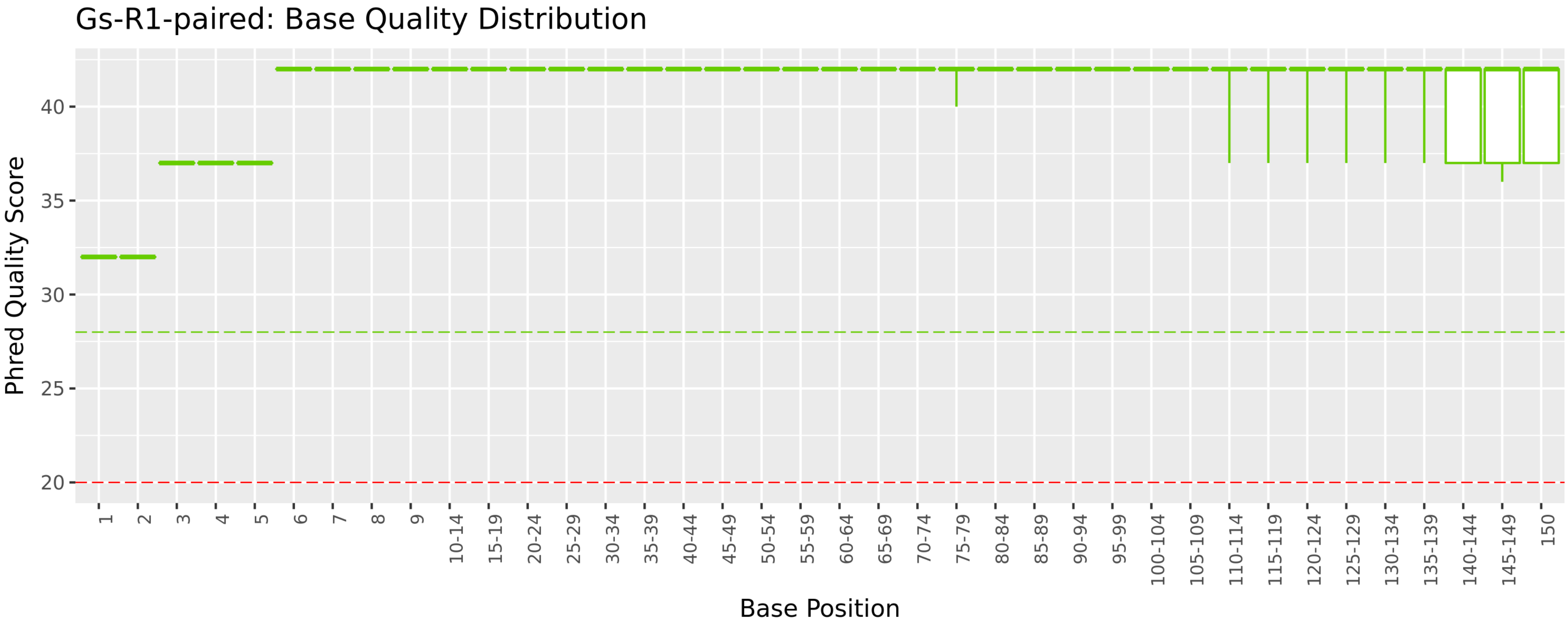

Gs-R1-paired: Base Quality Distribution
Phred Quality Score
40
35
30
25
20
1
2
3
4
5
6
7
8
9
10-14
15-19
20-24
25-29
30-34
35-39
40-44
45-49
50-54
55-59
60-64
65-69
70-74
75-79
80-84
85-89
90-94
95-99
100-104
105-109
110-114
115-119
120-124
125-129
130-134
135-139
140-144
145-149
150
Base Position

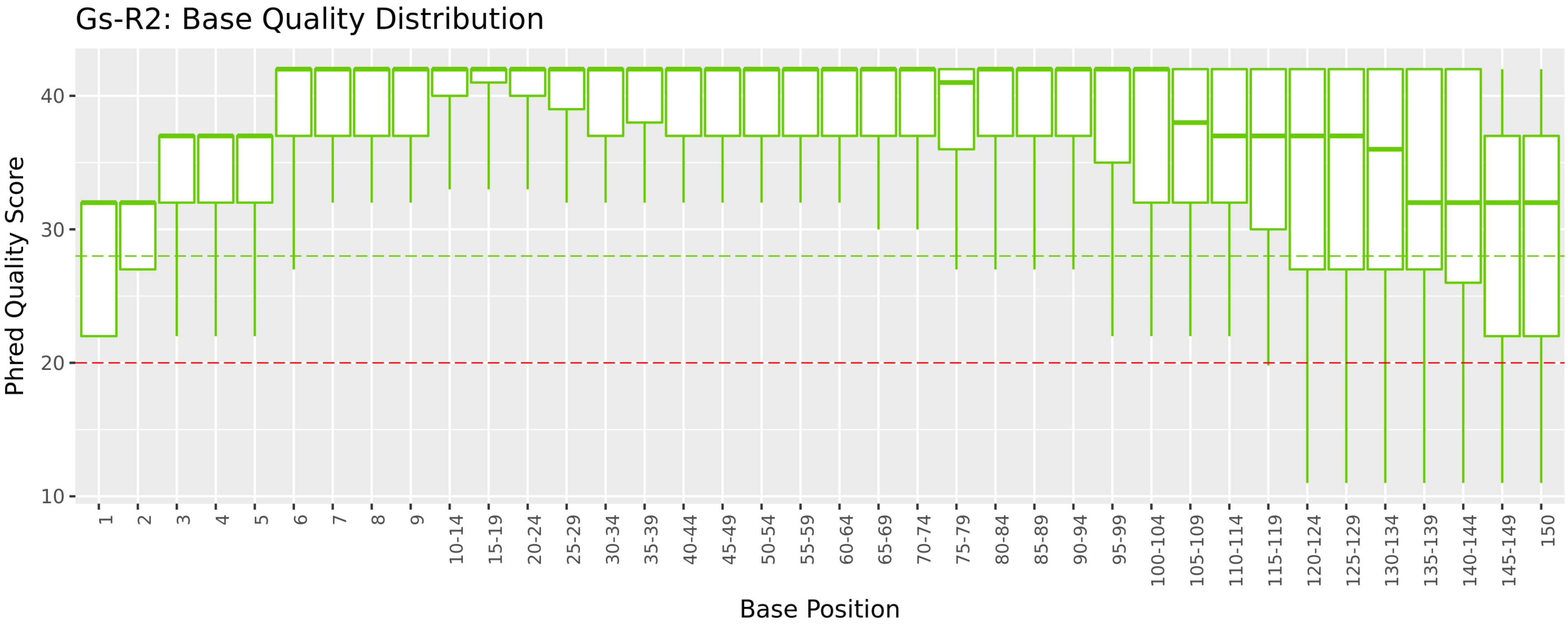

Gs-R2: Base Quality Distribution
Phred Quality Score
40
30
20
10
1
2
3
4
5
6
7
8
9
10-14
15-19
20-24
25-29
30-34
35-39
40-44
45-49
50-54
55-59
60-64
65-69
70-74
75-79
80-84
85-89
90-94
95-99
100-104
105-109
110-114
115-119
120-124
125-129
130-134
135-139
140-144
145-149
150
Base Position

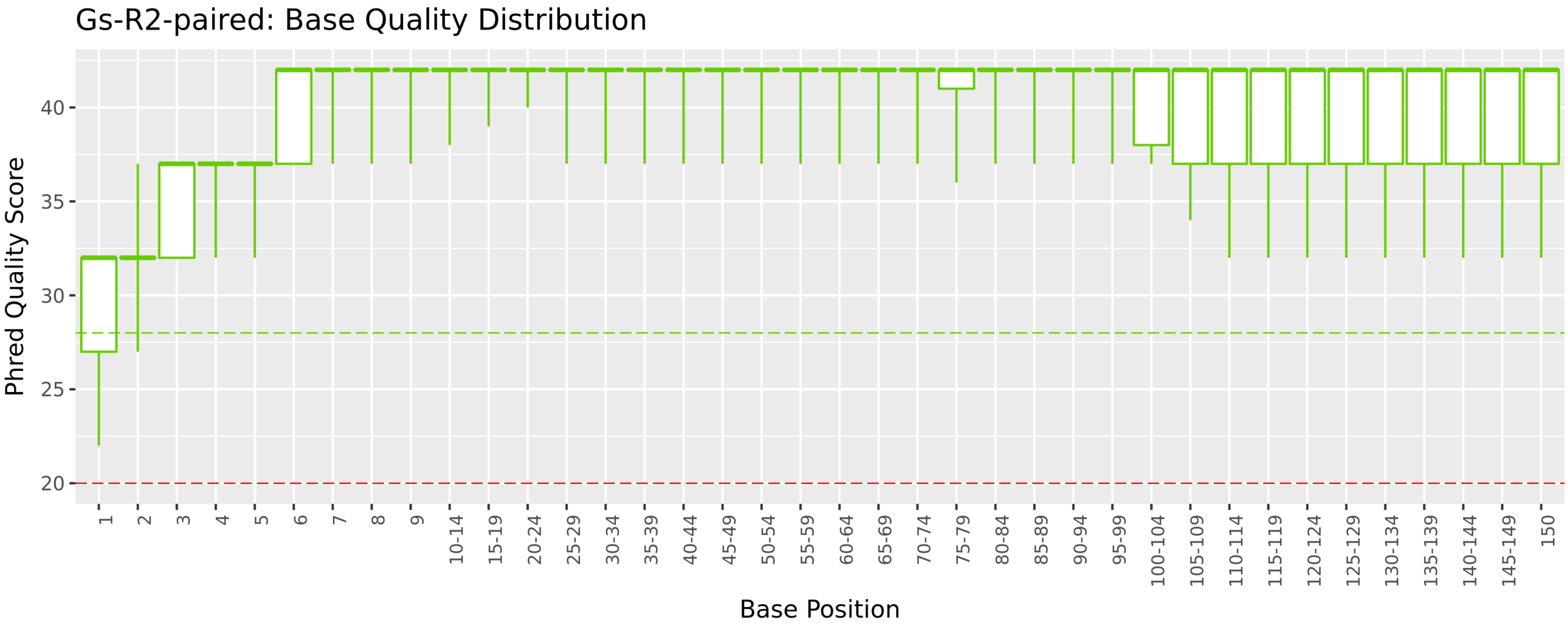
Gs-R2-paired: Base Quality Distribution
Phred Quality Score
40
35
30
25
20
1
2
3
4
5
6
7
8
9
10-14
15-19
20-24
25-29
30-34
35-39
40-44
45-49
50-54
55-59
60-64
65-69
70-74
75-79
80-84
85-89
90-94
95-99
100-104
105-109
110-114
115-119
120-124
125-129
130-134
135-139
140-144
145-149
150
Base Position
