# Supplementary material for: Transcriptome and excretory–secretory proteome of infective-stage larvae of the nematode Gnathostoma spinigerum reveal potential immunodiagnostic targets for development
Source: Parasite. 2019 Jun 5;26:34. doi: 10.1051/parasite/2019033 (PMC6550564; doi:10.1051/parasite/2019033)
Supplement: Supplementary file 5 — Supplementary Figure S4: Density (smoothed frequency) of the contigs according to the length of the advanced third-stage larvae of the Gnathostoma spinigerum (aL3Gs) data set (PDF 145 KB). [file parasite-26-34-s8.pdf]

# Supplementary Figure S4

Density (smoothed frequency) of the contigs according to the length of the advanced third-stage larvae of *Gnathostoma spinigerum* (aL3Gs) data set

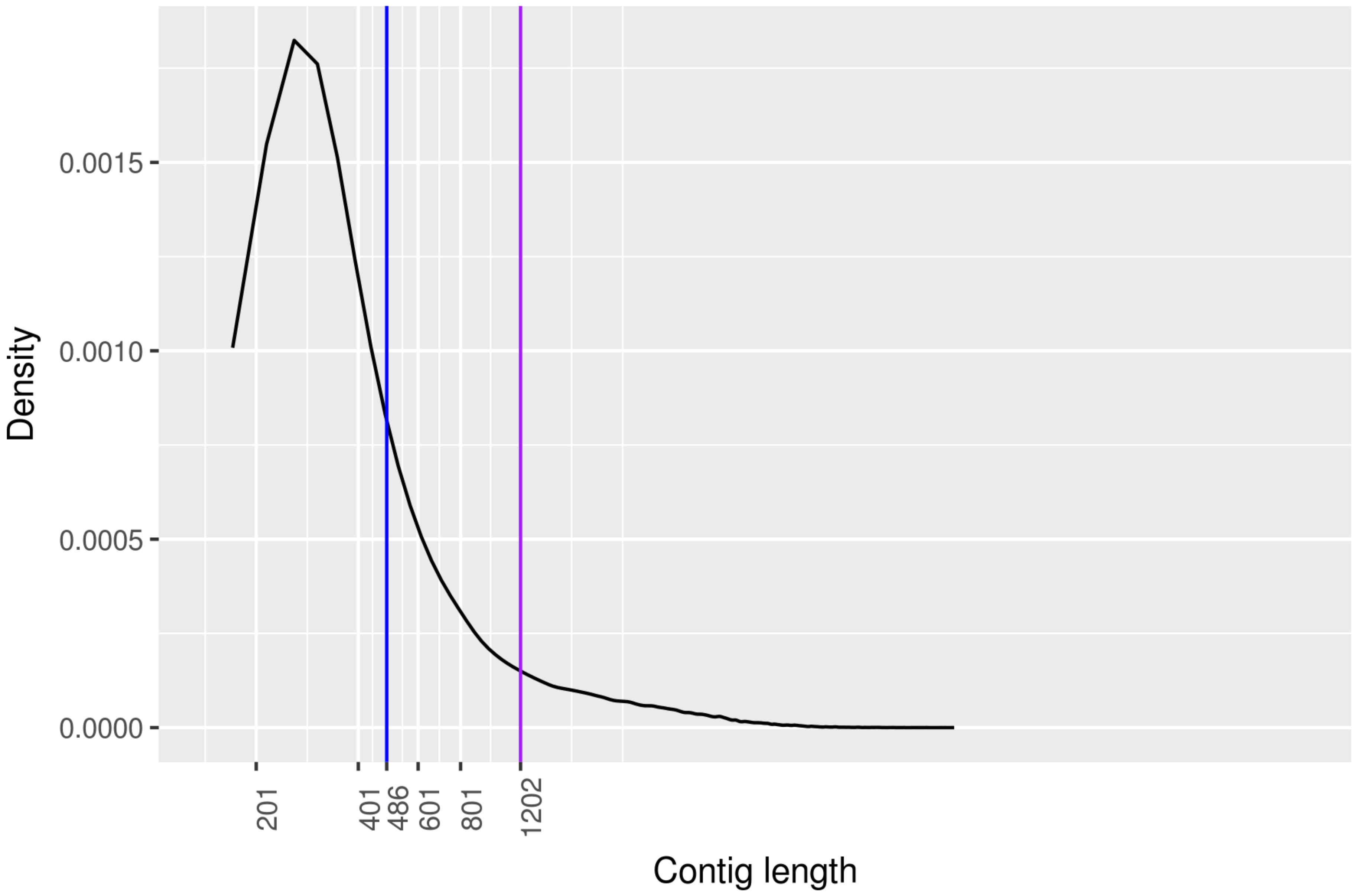

Density
0.0015
0.0010
0.0005
0.0000
201
401
486
601
801
1202
Contig length
