# Supplementary material for: Transcriptome and excretory–secretory proteome of infective-stage larvae of the nematode Gnathostoma spinigerum reveal potential immunodiagnostic targets for development
Source: Parasite. 2019 Jun 5;26:34. doi: 10.1051/parasite/2019033 (PMC6550564; doi:10.1051/parasite/2019033)
Supplement: Supplementary file 6 — Supplementary Figure S5: Species distribution of BLAST hits against the UniProt database (PDF 212 KB). [file parasite-26-34-s9.pdf]

Supplementary Figure S5

Species distribution of BLAST hits against the UniProt database

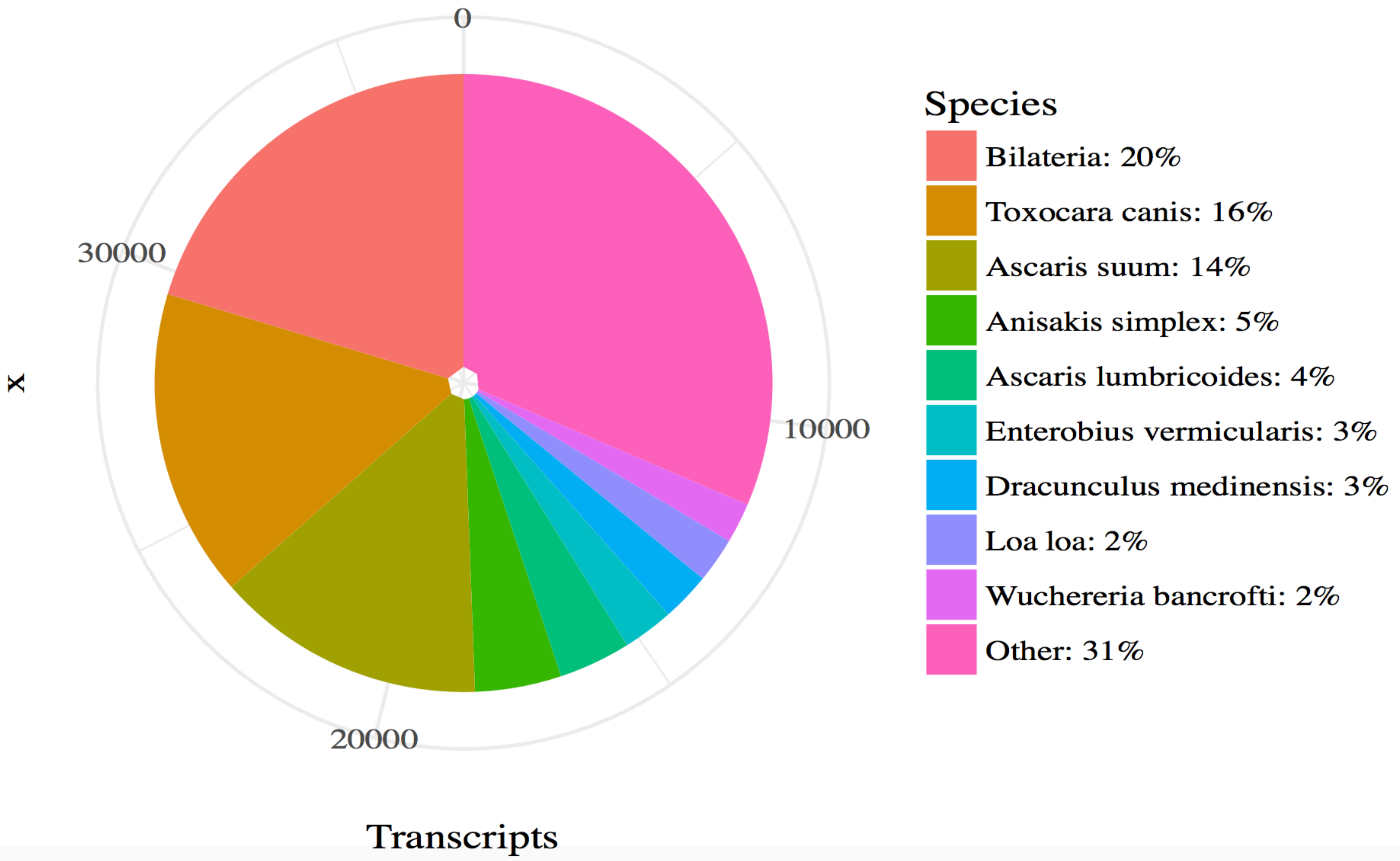

The pie chart depicts the species distribution of the best alignment, with a cutoff threshold of $1E^{-5}$.
