# Supplementary material for: Transcriptome and excretory–secretory proteome of infective-stage larvae of the nematode Gnathostoma spinigerum reveal potential immunodiagnostic targets for development
Source: Parasite. 2019 Jun 5;26:34. doi: 10.1051/parasite/2019033 (PMC6550564; doi:10.1051/parasite/2019033)
Supplement: Supplementary file 8 — Supplementary Figure S7: Top 20 enriched gene ontology (GO) terms among advanced third-stage larvae of Gnathostoma spinigerum (aL3Gs) transcripts (PDF 233 KB). [file parasite-26-34-s11.pdf]

Supplementary Figure S7

The top 20 enriched GO terms among advanced third-stage larvae of *Gnathostoma spinigerum* (aL3Gs) transcripts.

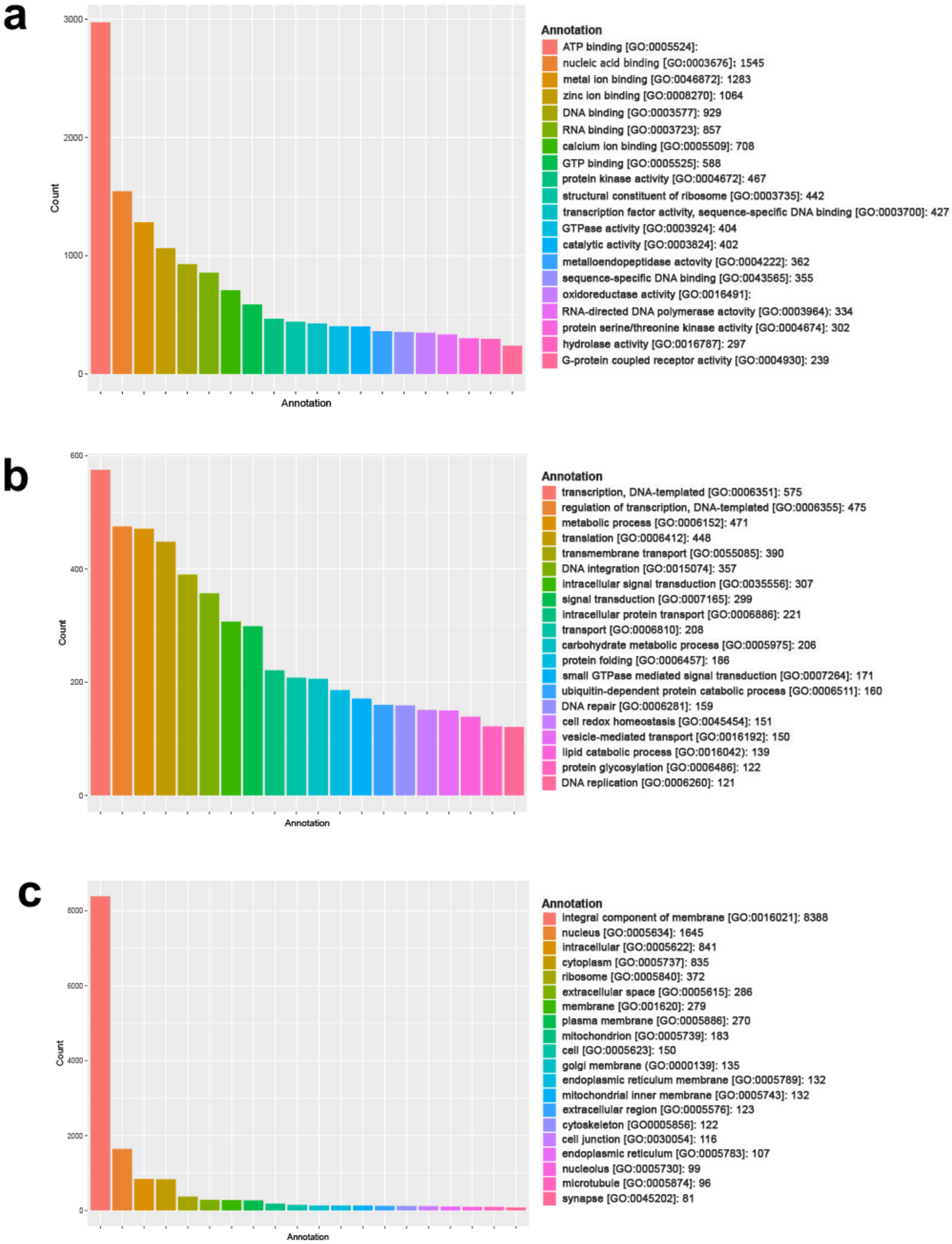

The transcript counts for the GO categories of molecular functions (a), cellular components (b), and biological processes (c). GO functions are shown on the right Y-axis. The number of transcripts with each GO function is shown on the left Y-axis.
